# Instability of LLM text embeddings for unsupervised dimension reduction of tabular data

**DOI:** 10.3389/fbinf.2026.1851023

**Published:** 2026-07-27

**Authors:** Jun Li, Yixuan Gou, Shawn Su, Mujun Xu, Brendan Chen

**Affiliations:** 1 Department of Applied and Computational Mathematics and Statistics, University of Notre Dame, Notre Dame, IN, United States; 2 The Awty International School, Houston, TX, United States; 3 The John Cooper School, The Woodlands, TX, United States; 4 Round Rock High School, Round Rock, TX, United States; 5 Shanghai American School Puxi, Shanghai, China

**Keywords:** biomedical data, dimension reduction, large language models, missing data, tabular data, text embeddings, unsupervised learning

## Abstract

Large language model (LLM) text embeddings have recently been used for supervised learning on tabular data by serializing each observation into text and then converting the text into a dense fixed-length vector. This strategy is appealing for biomedical tabular data, which are often mixed-type and contain missing values, because it produces a complete numeric representation even when some original entries are missing. However, its suitability for unsupervised tasks remains unclear. Here we evaluate the use of LLM-derived text embeddings for dimension reduction of tabular data, focusing on biological and clinical datasets. We compare an LLM embedding-based approach with a direct tabular approach that computes dissimilarities directly from the original variables. Because unsupervised dimension reduction has no ground-truth low-dimensional target, we assess performance through stability under perturbation. Across multiple datasets and analysis settings, the LLM embedding-based approach is consistently less stable than the direct tabular approach. In particular, small amounts of additional missingness and random permutation of feature order can substantially alter the resulting low-dimensional representation. These results suggest that the straightforward use of LLM text embeddings is not reliable for unsupervised dimension reduction of tabular data.

## 1 Introduction

The past decade has witnessed the remarkable success of deep neural networks across a wide range of applications. Their ability to learn from text, images, videos, and audio has transformed many areas of science and technology. In biological and medical research, deep learning has already been widely applied to problems such as medical imaging, analysis of text in medical records, and modeling of DNA, RNA, and protein sequences (Thirunavukarasu et al., 2023). More recently, the development of the transformer architecture and large language models (LLMs) has generated substantial interest in extending these advances to biological and medical data analysis.

There are different ways to leverage LLMs in biology and medicine. One strategy is to train transformer-based models or foundation models directly on domain-specific data. For example, foundation models have been trained on large collections of single-cell RNA sequencing data to learn cellular and genetic representations (Cui et al., 2024), and related efforts have also been made for electronic health records. While such approaches can be powerful, they typically require massive datasets, substantial computational resources, and considerable technical effort, making them inaccessible to many research groups.

A second strategy is to use a general-purpose LLM, such as ChatGPT, directly as a chatbot or to build agent-like systems from it. In this setting, the user provides a prompt and the model generates a response in natural language. For example, GPTcelltype (Hou and Ji, 2024) uses ChatGPT as a single-prompt annotator for single-cell data: given a list of marker genes for a cell cluster, ChatGPT is asked to directly predict the cell type. This strategy is often simple and intuitive, but its output is usually qualitative, prompt-dependent, and not always easy to integrate into standard quantitative pipelines.

A third strategy, which is the focus of this paper, is to use the text-embedding functionality of general-purpose LLMs. For example, OpenAI provides embedding models through its API (https://developers.openai.com/api/docs/guides/embeddings) that map an arbitrary text input—a word, a sentence, a paragraph, or even a longer document—to a dense fixed-length numeric vector. Importantly, the length of the output vector is fixed for a given embedding model, regardless of the length of the input text. For instance, the text-embedding-3-small model returns a vector of length 1,536, whereas the text-embedding-3-large model returns a vector of length 3,072. These vectors are intended to capture information contained in the input text, especially its semantic content, and can then be used as quantitative representations for downstream analysis. Compared with training a domain-specific transformer from scratch, this strategy is far easier to use. Compared with using an LLM as a chatbot, it produces numeric outputs that can be directly incorporated into standard statistical and machine learning workflows.

Although this text embedding-based strategy is less widely known in biological and medical research than the previous two strategies, it is becoming increasingly popular because of its simplicity, accessibility, and quantitative nature. For example, GenePT converts NCBI gene descriptions into embeddings and uses them for downstream prediction tasks such as gene property and cell-type prediction (Chen and Zou, 2025). Similar ideas have also been applied to problems such as modeling gene perturbation outcomes (e.g., our own prior work (Zhu and Li, 2026)), integrating gene-related representations into multimodal biomedical frameworks (Liu et al., 2026; Chen et al., 2025a; Liu et al., 2025; Chen et al., 2025b), and representing clinical concepts in electronic health records for disease risk prediction (Park et al., 2025; Yao et al., 2025).

However, text embedding-based methods require the input to be text. This raises a natural question for one of the most common forms of data in biology and medicine: tabular data. Biological and clinical studies frequently produce tabular datasets, including patient demographics, laboratory measurements, survey variables, clinical covariates, and other structured metadata. Such data are often heterogeneous, containing a mixture of continuous, categorical, ordinal, and binary variables. They also very often contain missing values, sometimes at substantial levels. These characteristics make tabular data both ubiquitous and challenging, especially for downstream analyses that depend on defining meaningful similarity or distance between observations.

A natural idea is therefore to convert each tabular observation into a text description and then use an LLM embedding model to obtain a numeric representation of that observation. For example, a row of tabular data might be serialized into text such as “Age: 27, Gender: Female, Smoking: missing.” This text can then be passed to an embedding model, which produces a fixed-length numeric vector. Repeating this procedure for all observations yields a new data matrix consisting of embeddings, which can then be used in place of the original tabular data for downstream machine learning tasks. Because the output embeddings are dense fixed-length vectors, this approach provides an apparently simple and clean way to convert mixed-type tabular data into a uniform numeric representation.

This strategy has recently been studied mainly for supervised learning tasks such as classification and regression (Dinh et al., 2022; Hegselmann et al., 2023; Jiang et al., 2026; Hollmann et al., 2022). Although it does not consistently outperform strong traditional methods such as tree-based models on clean and complete datasets, it has shown promise in certain settings, especially when sample sizes are small or missingness is substantial. These findings make the approach appealing. Indeed, once the original tabular data have been converted into embeddings, one obtains a new numeric data matrix that can, at least in principle, be used as the input for many standard machine learning procedures.

In this paper, we ask whether this appealing strategy also works well for *unsupervised* dimension reduction. This question is especially important for tabular biomedical data with missing values. Dimension reduction methods typically rely on pairwise distances or dissimilarities, but defining such quantities for incomplete mixed-type tabular data is often nontrivial. In contrast, the LLM embedding-based approach appears to bypass this difficulty in a straightforward way: even if some entries in the original tabular observation are missing, the serialized text can still be embedded into a complete dense vector. For example, text such as “Age: missing, Gender: Male” still yields a fixed-length numeric embedding. As a result, the method seems to offer a particularly convenient solution for dimension reduction of incomplete tabular data.

However, good performance in supervised prediction does not necessarily imply suitability for unsupervised analysis. In supervised learning, downstream labels may help the learning algorithm focus on task-relevant variation while ignoring nuisance variation in the representation. In unsupervised dimension reduction, by contrast, the representation itself determines the geometry of the data. If the embedding process introduces artificial variation that is unrelated to the intrinsic relationships among observations, the resulting low-dimensional representation may become unstable or misleading.

Motivated by these considerations, we systematically study the use of LLM-derived text embeddings for dimension reduction of mixed-type tabular data, with particular emphasis on biological and clinical datasets containing missing values. We compare the LLM embedding-based approach with a direct tabular approach, in which dissimilarities are computed directly from the original tabular variables without introducing an intermediate embedding representation. Our evaluation focuses on stability under perturbations that should ideally have limited impact on the underlying geometry, including perturbations due to missingness and permutations of feature order. Across multiple datasets and analysis settings, we find that the LLM embedding-based approach is substantially less stable than the direct tabular approach. These results suggest that, despite its convenience and intuitive appeal, the LLM embedding-based approach may be poorly suited for unsupervised dimension reduction of tabular data.

## 2 Methods

### 2.1 LLM embedding-based approach

**FIGURE 1:**
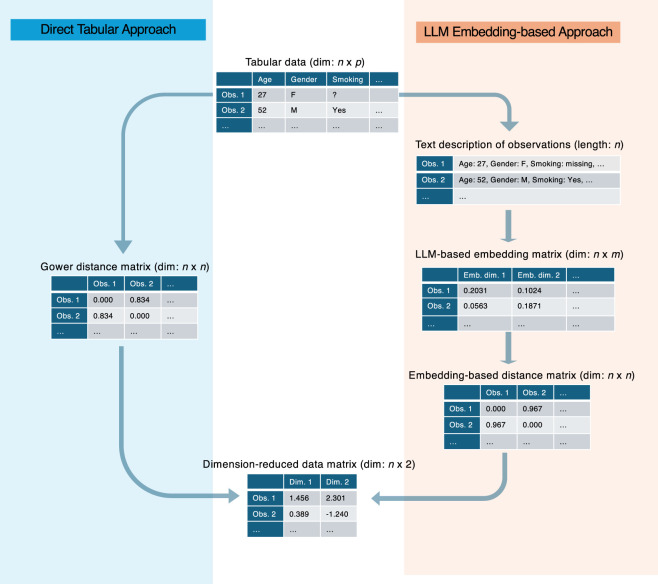
Direct tabular vs. LLM embedding-based approaches for dimension reduction of tabular data. In the direct tabular approach, pairwise dissimilarities are computed directly from the original tabular data (e.g., using the Gower distance), and the resulting distance matrix is used as input to a dimension reduction method to obtain a low-dimensional representation. In contrast, the LLM embedding-based approach first converts each observation into a text description using a predefined template, and then applies an LLM embedding model to obtain a vector representation. These embeddings are treated as a new data matrix, from which pairwise distances are computed and subsequently used for dimension reduction.

We evaluated an LLM embedding-based approach for dimension reduction of mixed-type tabular data with missing values. As illustrated in the right panel of Figure 1, the pipeline consists of four main steps: text serialization, embedding generation, distance matrix calculation, and dimension reduction.

First, each observation (row) in the tabular dataset was serialized into a single text string using a simple template. Specifically, feature names and feature values were connected by colons, and different feature–value pairs were separated by commas. For example, an observation with Age = 27, Gender = F, and Smoking = missing was converted to the text “Age: 27, Gender: F, Smoking: missing.” Missing values were therefore represented explicitly in the serialized text. In the main analyses, we used this colon-based serialization scheme throughout. Previous studies on supervised learning with serialized tabular data (e.g. (Hegselmann et al., 2023)) have also considered alternative templates, such as using the word “is” to connect a feature and its value, and reported performance similar to that of the colon-based template used here.

Second, the serialized text strings were passed to the OpenAI embedding API using the text-embedding-3-small model, which returns a dense numeric vector of length 1,536 for each input text. The output dimension is fixed for this model and does not depend on the length or content of the input text.

Third, the resulting embedding vectors were treated as the new representation of the observations, and a pairwise distance matrix was computed from these embeddings. Unless otherwise specified, cosine distance was used, as it is OpenAI’s recommended choice (OpenAI, 2024).

Finally, the distance matrix was used as input to two dimension reduction methods: classical multidimensional scaling (MDS) (Torgerson, 1952; Cox and Cox, 2008) and Uniform Manifold Approximation and Projection (UMAP) (McInnes and Healy, 2018). We chose these two methods because both can operate on an arbitrary pairwise distance matrix, which is central to our comparison framework. In addition, they represent two distinct types of dimension reduction. Classical MDS is deterministic once the distance matrix is fixed and can be obtained by eigen-decomposition, whereas UMAP is a nonlinear method that relies on stochastic optimization. This distinction is important for interpreting the stability results presented later. We did not use principal component analysis (PCA), because it operates directly on a complete numeric data matrix rather than on an arbitrary pairwise distance matrix, making it less suitable for comparing distance-based approaches for mixed-type tabular data with missing values.

### 2.2 Direct tabular approach

We compared the LLM embedding-based approach with a direct tabular approach, in which dissimilarities are computed directly from the original tabular variables without introducing an intermediate embedding representation. This procedure is illustrated in the left panel of Figure 1. Specifically, we first computed a pairwise distance matrix from the original tabular data using a distance measure designed for mixed-type variables and missing values, and then applied MDS or UMAP to that distance matrix.

As the primary baseline, we used the Gower distance (Gower, 1971), which is specifically designed for mixed-type data and is widely used in exploratory analysis and clustering (Kaufman and Rousseeuw, 2009). The Gower distance defines dissimilarity as an average of feature-wise contributions, with continuous variables contributing scaled absolute differences and categorical variables contributing mismatch indicators. Missing values are handled through pairwise omission, so that each pairwise distance is computed using only the features observed for both observations. When two observations share no comparable features, the distance is set to its maximum value in our implementation. More details are provided in the Supplementary Material.

In addition to the Gower distance, we also considered two alternative distance measures for mixed-type tabular data with missing values: a NaN-aware Euclidean distance (Dixon, 2007) and the Heterogeneous Euclidean-Overlap Metric (HEOM) (Randall Wilson and Martinez, 1997). The NaN-aware Euclidean distance first transforms all variables into a unified numeric representation and then computes a rescaled Euclidean distance using only the dimensions observed for both observations. HEOM instead aggregates feature-wise dissimilarities directly and treats missing values as maximally dissimilar. Full definitions of these distance measures and their corresponding results are provided in the Supplementary Material and briefly discussed in the Conclusions and Discussion section.

### 2.3 Data

To evaluate the methods across a range of realistic settings, we used multiple real-world tabular datasets from biological and clinical research. These datasets span a broad range of characteristics: some are fully numeric with no missing values, while others are predominantly categorical with more than 20% missingness. Sample sizes range from around 150 to 1,000 observations, and the number of features ranges from 12 to 63. For datasets with more than 1,000 observations, a random subsample of 1,000 observations was used. Standard preprocessing was applied to remove administrative identifiers and wholly unstructured free-text fields, while retaining the structured continuous and categorical variables that are typical of biomedical tabular data. Detailed descriptions of the datasets and preprocessing steps are provided in the Supplementary Material. A summary of the eight datasets, including sample size, number of features, percentage of missing values, and proportion of categorical features, is given in Table 1.

**TABLE 1:** Summary of the datasets used in the evaluation, including sample size, number of features, percentage of missing values, percentage of categorical features, and corresponding references.

Dataset	Samples	Features	Missing (%)	Categorical (%)	References
Parkinsons	195	22	0.00	0.00	Tsanas et al. (2009)
WDBC	569	30	0.00	0.00	Nick Stre et al. (1993)
HCV	615	12	0.42	8.33	Hoffmann et al. (2018)
MIMIC-IV	140	63	5.31	14.29	Johnson et al. (2023), Goldberger et al. (2000)
eICU	1,000	53	9.53	16.98	Goldberger et al. (2000), Johnson et al. (2021)
NHANES	1,000	36	20.28	44.44	Centers for Disease Control and Prevention CDC (2020), Randall (2014)
Hepatitis	155	19	5.67	68.42	Hepatitis (1983)
Thyroid-diff	383	16	0.00	93.75	Borzooei et al. (2024)

## 3 Results

### 3.1 Stability-based evaluation in the unsupervised setting

Evaluating dimension reduction methods is inherently challenging in a fully unsupervised setting, because there is typically no ground-truth low-dimensional representation against which the result can be directly compared. In this work, our central question is whether the LLM embedding-based approach yields a stable low-dimensional representation of tabular data. We therefore evaluate performance through *stability under perturbation*. In particular, if a dimension reduction pipeline is suitable for tabular data with missing values, then introducing a small amount of additional missingness should not substantially alter the resulting low-dimensional representation.

Let $Z={\left(Z_{1},\ldots ,Z_{n}\right)}^{⊤}\in R^{n\times d}$ denote the low-dimensional representation obtained from the original data, and let $Z^{\prime }={\left(Z_{1}^{\prime },\ldots ,Z_{n}^{\prime }\right)}^{⊤}\in R^{n\times d}$ denote the representation obtained after a perturbation of the input data, such as the introduction of additional missingness or a permutation of feature order. To quantify the difference between Z and $Z^{\prime }$ , one cannot directly use the Frobenius norm $\|Z-Z^{\prime }{\|}_{F}^{2}$ , because low-dimensional embeddings are generally identifiable only up to translation, rotation, and reflection, and in some cases uniform scaling. Two practically equivalent embeddings may therefore differ substantially in raw coordinates.

We therefore compare Z and $Z^{\prime }$ using three complementary similarity measures. The first is Procrustes similarity, which we define as one minus the Procrustes discrepancy (Gower, 1975). Specifically, $Z^{\prime }$ is aligned to Z by allowing translation, rotation (and reflection), and uniform scaling. That is, an aligned version ${\tilde{Z}}^{\prime }$ is obtained by minimizing

$$\|Z-\left(sZ^{\prime }R+1b^{⊤}\right){\|}_{F}^{2}$$

over a scalar s>0 , an orthogonal matrix R satisfying $R^{⊤}R=I$ , and a translation vector $b\in R^{d}$ . The resulting minimized discrepancy quantifies the difference in overall geometric configuration after optimal alignment. In practice, we compute this quantity using the procrustes function in the Python package scipy.spatial, which returns a discrepancy value between 0 and 2 (SciPy Community, 2026). We then define Procrustes similarity as one minus this discrepancy, so that larger values indicate greater similarity.

The second is KNN similarity. For each observation i , let $N_{K}\left(i\right)$ and $N_{K}^{\prime }\left(i\right)$ denote its sets of K nearest neighbors in Z and $Z^{\prime }$ , respectively. We quantify neighborhood preservation by the Jaccard index

$$J_{K}\left(i\right)=\frac{\left|N_{K}\left(i\right)\cap N_{K}^{\prime }\left(i\right)\right|}{\left|N_{K}\left(i\right)\cup N_{K}^{\prime }\left(i\right)\right|},$$

and then average this quantity over all observations. By default, we use K=3 . This measure primarily reflects preservation of local structure.

The third is KMeans similarity. To assess coarse-grained structural consistency, we apply K -means clustering with a fixed number of clusters to both Z and $Z^{\prime }$ , and then compare the resulting partitions using the Adjusted Rand Index (ARI). By default, we use K=3 . This measure reflects agreement in coarse global structure.

All three measures take larger values when the two low-dimensional representations are more similar, with 1 indicating perfect agreement. In our setting, values close to 0 indicate essentially no meaningful agreement: for Procrustes similarity, little global geometric agreement after alignment; for KNN similarity, little preservation of local neighborhoods; and for KMeans similarity, little consistency in coarse cluster structure.

These measures capture complementary aspects of stability. Procrustes similarity reflects overall geometric agreement, KNN similarity emphasizes local neighborhood preservation, and KMeans similarity focuses on coarse-grained cluster structure. All stability measures were computed over five independent repetitions, and results are reported as means with standard errors.

### 3.2 Instability under missingness

#### 3.2.1 Missing completely at random (MCAR)

We first considered additional missingness introduced completely at random. Specifically, for each cell in the tabular data matrix, its value was set to missing independently with probability α , where α∈{0,0.02,0.1,0.2,0.5} . Here α=0 corresponds to no additional missingness, α=0.02 corresponds to a very small perturbation, and α=0.5 corresponds to an aggressive perturbation in which half of the cells are set to missing.

**FIGURE 2:**
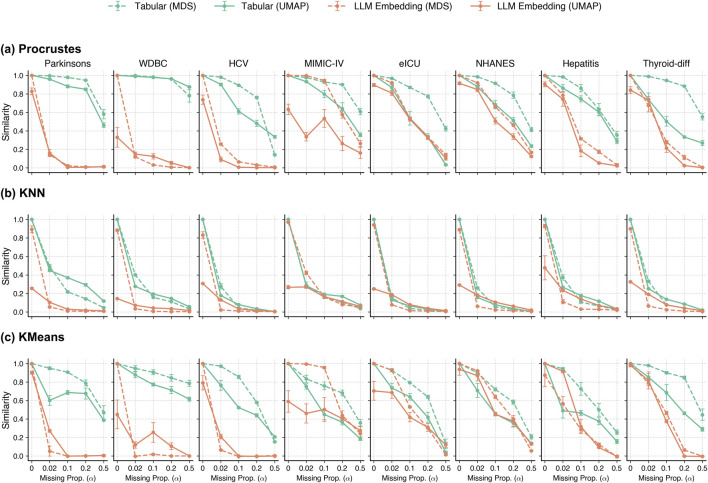
Dimension reduction stability under missing completely at random (MCAR). Additional missing values were introduced independently into each cell with probability α . Stability was assessed by comparing the resulting low-dimensional representation with that obtained from the original data using three complementary measures: **(a)** Procrustes similarity, **(b)** KNN similarity, and **(c)** KMeans similarity. Higher values indicate greater stability, with 1 corresponding to perfect agreement and values near 0 indicating little meaningful agreement. Results are shown for both the LLM embedding-based approach and the direct tabular approach, each combined with either MDS or UMAP.

The results for the three similarity measures are shown in Figure 2. Across datasets and across all three similarity measures, the same overall pattern is observed: the LLM embedding-based approach is substantially less stable than the direct tabular approach when additional missingness is introduced.

This difference is already evident under very small perturbations. Under α=0.02 , the LLM embedding-based approach often shows a marked drop in similarity, especially for datasets such as WDBC, Parkinsons, and HCV. Thus, even a small amount of additional missingness can substantially alter the low-dimensional representation produced by the LLM embedding-based pipeline.

Under more severe perturbations, this instability becomes even more pronounced. For example, as shown in Figure 2a, when α=0.5 , Procrustes similarity for the LLM embedding-based approach is below 0.2 for nearly all datasets and is close to zero for several of them, indicating near-complete loss of geometric agreement with the original low-dimensional representation. In contrast, the direct tabular approach remains substantially more stable across the same perturbation levels.

An additional and important observation is that, for the LLM embedding-based approach, similarity is sometimes noticeably below 1 even when α=0 , that is, when no additional missingness is introduced. In principle, one would expect identical input data to produce identical serialized text and hence identical embeddings. However, after systematic checking, we found that repeated calls to the OpenAI embedding API can yield slightly different embedding vectors even when the input text is completely identical. Comparing baseline embeddings from two independent runs, a substantial fraction of samples received numerically different vectors: for example, 65% in the HCV dataset and 32% in the eICU dataset. Because the embeddings are normalized to unit length, the magnitude of these differences can be directly interpreted. The median $\ell_{2}$ difference for affected samples was typically around 0.001, but the distribution was heavy-tailed, with the largest deviation reaching approximately 0.1 in some datasets. This effect is especially visible for UMAP, whose neighbor-graph construction depends on local distance rankings, so that even a small number of outlier perturbations can alter the nearest-neighbor structure and cascade into noticeably different low-dimensional projections. In contrast, MDS is much less affected because it is deterministic once the distance matrix is fixed.

This observation has important practical implications. Even if two researchers start from the same dataset, use the same text-serialization scheme, the same embedding model, and the same UMAP random seed, they may still obtain noticeably different low-dimensional representations because of upstream numerical stochasticity in the embedding API. This raises concerns about the reproducibility of the LLM embedding-based approach in unsupervised analysis.

The same qualitative conclusions are also supported by KNN similarity and KMeans similarity (Figures 2b,c). KNN similarity tends to be more sensitive to perturbation, which is expected because local neighborhood relationships can change even under relatively small geometric perturbations. KMeans similarity broadly tracks the behavior of Procrustes similarity, although with somewhat greater variability due to the additional randomness introduced by the clustering procedure.

#### 3.2.2 Missing not at random (MNAR)

We next considered a structured missing-not-at-random (MNAR) perturbation. Unlike the MCAR setting, in which any cell is equally likely to become missing, the MNAR mechanism targets specific subsets of observations within each feature. For numeric features, additional missingness was introduced only among observations falling below the feature median; for categorical features, only among observations belonging to the most frequent category. Within these targeted subsets, each cell was set to missing independently with probability α . This produces a more structured form of missingness than MCAR, because the probability of a value being missing now depends on the value itself.

**FIGURE 3:**
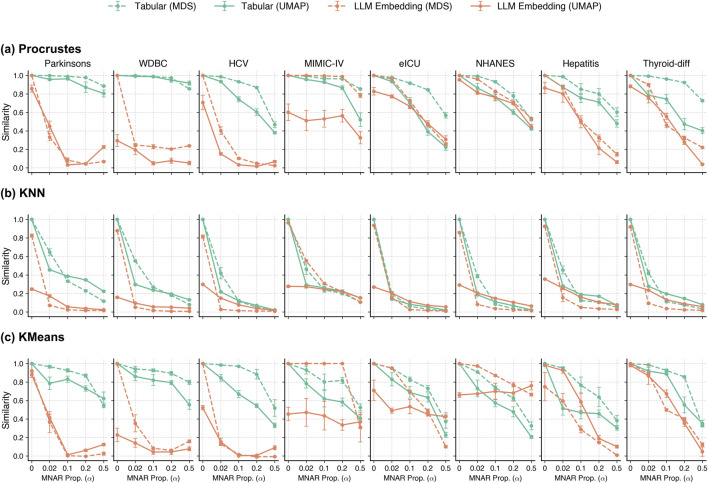
Dimension reduction stability under missing not at random (MNAR). Additional missing values were introduced using a structured missingness mechanism, and the resulting low-dimensional representation was compared with that from the original data. Stability was quantified using **(a)** Procrustes similarity, **(b)** KNN similarity, and **(c)** KMeans similarity. Higher values indicate greater stability, with 1 corresponding to perfect agreement and values near 0 indicating little meaningful agreement. Results are shown for both the LLM embedding-based approach and the direct tabular approach, each combined with either MDS or UMAP.

The results are shown in Figure 3. The same main conclusion holds: across datasets and similarity measures, the LLM embedding-based approach is consistently less stable than the direct tabular approach. The qualitative pattern is similar to that observed under MCAR, confirming that the instability of the LLM embedding-based pipeline is not restricted to purely random missingness but also persists under a structured missingness mechanism.

### 3.3 Instability under feature-order permutation

We next examined a second, conceptually distinct perturbation: random permutation of feature order. This issue is specific to the LLM embedding-based approach. In tabular data analysis, the columns of a data matrix generally have no intrinsic order. However, once an observation is serialized into text, the features appear in a particular sequence, and that sequence becomes part of the text presented to the embedding model. Ideally, the resulting low-dimensional representation should not depend strongly on such arbitrary feature ordering.

For the direct tabular approach, this invariance holds by construction: permuting the columns does not change the underlying tabular data values or the resulting distance matrix, and hence should not change the low-dimensional representation apart from numerical or downstream algorithmic variability. For the LLM embedding-based approach, however, the serialized text is order-sensitive, and so the generated embeddings may also become order-sensitive.

**FIGURE 4:**
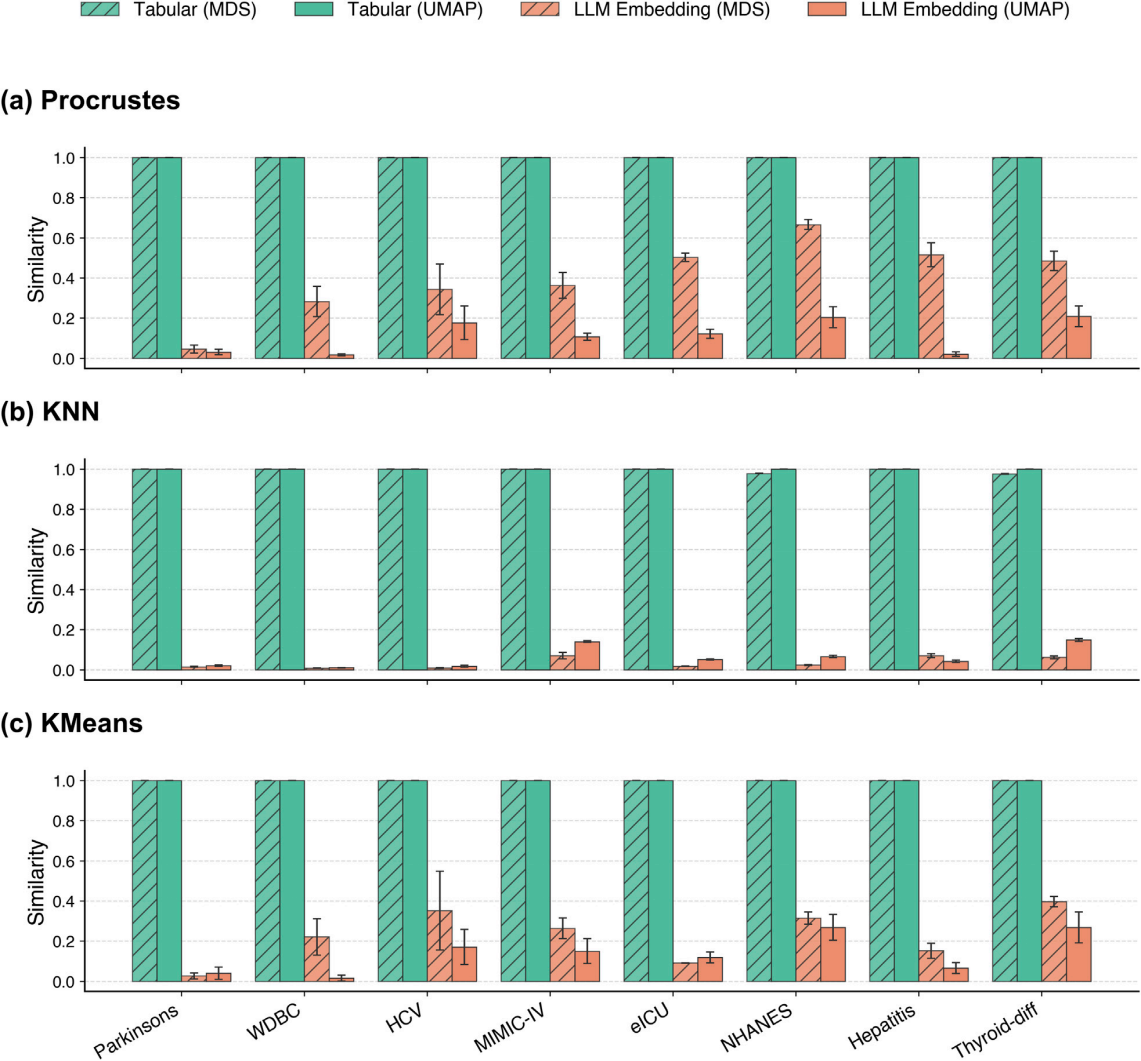
Dimension reduction stability under permutation of feature order. For each dataset, the columns of the tabular data were randomly permuted, and the resulting low-dimensional representation was compared with that obtained from the original unpermuted data. Stability was quantified using **(a)** Procrustes similarity, **(b)** KNN similarity, and **(c)** KMeans similarity. Higher values indicate greater stability, with 1 corresponding to perfect agreement and values near 0 indicating little meaningful agreement. Results are shown for both the LLM embedding-based approach and the direct tabular approach, each combined with either MDS or UMAP.

To evaluate this effect, we randomly permuted the feature order, recomputed the low-dimensional representation using both approaches, and compared the result with that obtained from the original unpermuted data. The three similarity measures are shown in Figure 4.

The direct tabular approach remains essentially invariant under this perturbation, as expected: all three similarity measures are at or very near 1.00 for every dataset. In contrast, the LLM embedding-based approach shows a dramatic loss of stability. Under Procrustes similarity, the average across datasets is approximately 0.40 for MDS and 0.11 for UMAP, indicating that even overall geometric structure is poorly preserved. KNN similarity is lower still, averaging approximately 0.03 for MDS and 0.06 for UMAP, meaning that local neighborhood structure is almost entirely disrupted by feature-order permutation. KMeans similarity shows a similar pattern, averaging approximately 0.23 for MDS and 0.14 for UMAP.

This result is particularly important because feature order in tabular data is usually arbitrary and should not carry substantive information. The strong sensitivity of the LLM embedding-based approach to feature permutation therefore indicates that the embedding process incorporates artificial ordering information that is not intrinsic to the data itself. In an unsupervised dimension-reduction setting, where the learned representation directly determines the geometry of the output, such artificial structure can substantially distort the resulting low-dimensional embedding.

### 3.4 Robustness analyses

We carried out a range of additional analyses to assess the robustness of the findings reported above.

#### 3.4.1 Alternative text-serialization templates

In all main analyses, each tabular observation was serialized using a colon-based template (e.g., “Age: 27, Gender: F”). We repeated the entire set of experiments using two alternative templates. The first is an “is”-based template (e.g., “Age is 27. Gender is F.“), which changes both the delimiter tokens and the sentence structure presented to the embedding model. The results (Supplementary Figures S13–S15) are qualitatively indistinguishable from those obtained with the colon template. The second is a JSON-based template (e.g.,:{“Age”: 27, “Gender”: “F”}::), which uses a structurally distinct serialization format widely used in data interchange. The results (Supplementary Figures S16–S18) again show the same patterns of instability under both missingness perturbations and feature-order permutation. Across all three serialization strategies, the LLM embedding-based approach remains substantially less stable than the direct tabular approach, suggesting that the observed instability is not an artifact of a particular serialization template but reflects a more fundamental property of the text-serialization-then-embedding pipeline.

#### 3.4.2 Deterministic open-source embedding model

The main analyses used OpenAI’s embedding API, which is nondeterministic: repeated calls with identical input can yield slightly different embedding vectors, as noted in Section 3.2.1. To determine whether this API nondeterminism contributes to the observed instability, we repeated all experiments using all-mpnet-base-v2 (Song et al., 2020), a high-performing open-source embedding model from the Sentence-Transformers library (Reimers and Gurevych, 2019) that runs locally and produces fully deterministic outputs. This model was selected because of its strong performance on semantic textual similarity benchmarks. It differs from the OpenAI models in architecture (MPNet rather than GPT), dimensionality (768 rather than 1,536 or 3,072), and provenance (UKP Lab, TU Darmstadt). The results (Supplementary Figures S19–S21) show that the same patterns of instability under missingness persist with the deterministic model, confirming that the instability is an inherent property of the text-serialization-then-embedding pipeline rather than an artifact of API nondeterminism.

#### 3.4.3 Canonical feature ordering

In the main analyses, features were serialized in their original column order, and the permutation experiments showed that arbitrary reordering can substantially alter the resulting embeddings. A natural mitigation is to sort the features into a canonical order before serialization. We repeated the missingness experiments after sorting columns alphabetically by feature name, so that the same canonical ordering is applied regardless of the original column arrangement (Supplementary Figures S22–S23). However, the same dataset may admit multiple equally valid canonical orderings (e.g., the original column order, alphabetical order, grouping by data type), and these generally produce very different embeddings and downstream results, making it unclear which canonical ordering should be preferred. Moreover, regardless of which canonical ordering is used, the instability under missingness remains unchanged.

#### 3.4.4 Larger embedding model

We also repeated all experiments using OpenAI’s larger embedding model, text-embedding-3-large, which produces vectors of dimension 3,072 (compared with 1,536 for text-embedding-3-small). The results (Supplementary Figures S24–S26) show that the larger model does not meaningfully improve stability. The same patterns of instability under missingness and feature-order permutation persist, indicating that the issue is not simply one of embedding capacity.

#### 3.4.5 Alternative distance measures on the embeddings

In the main analyses, cosine distance was used to compute pairwise distances between embedding vectors, as recommended by OpenAI. We also considered Euclidean distance (Supplementary Figures S1–S3) and standardized Euclidean distance (Supplementary Figures S4–S6). The main conclusions remained unchanged under both alternatives.

#### 3.4.6 Alternative direct-tabular distance measures

For the direct tabular approach, the main analyses used the Gower distance. We also considered two alternative distance measures for mixed-type tabular data with missing values: the NaN-aware Euclidean distance (Supplementary Figures S7–S9) and the Heterogeneous Euclidean-Overlap Metric (HEOM; (Supplementary Figures S10–S12). Both alternatives confirmed the same pattern: the direct tabular approach remains substantially more stable than the LLM embedding-based approach.

#### 3.4.7 Different values of K

We varied the parameter K used in the KNN similarity and KMeans similarity measures, considering K=2 (Supplementary Figures S27–S29) and K=5 (Supplementary Figures S30–S32) in addition to the default K=3 . The qualitative conclusions were unchanged across all values of K .

Taken together, these analyses indicate that the central findings of this paper—the instability of the LLM embedding-based approach under missingness and feature-order permutation—are robust across three embedding models, three serialization templates, canonical feature ordering, multiple distance measures, and different parameter values, and are not driven by any single implementation choice.

## 4 Conclusions and discussion

In this paper, we studied a natural extension of recent LLM-based tabular learning ideas to unsupervised dimension reduction. The approach is appealing for several reasons. By serializing each tabular observation into text and then embedding that text into a fixed-length numeric vector, it provides a simple and accessible way to convert mixed-type tabular data into a uniform numeric representation. In particular, the approach appears especially attractive for incomplete tabular data, because missing values in the original data do not prevent the embedding model from producing a complete dense vector.

Our results, however, show that this straightforward LLM embedding-based approach is not reliable for unsupervised dimension reduction of tabular data. The first major finding is its instability under missingness. Even a small amount of additional missingness can substantially alter the resulting low-dimensional representation, affecting both global geometry and local neighborhood structure. This suggests that, despite its apparent convenience, the approach should be used with considerable caution when missing data are present. Moreover, even in the absence of any additional missingness, the non-determinism of the embedding API itself can produce noticeably different low-dimensional representations across independent runs, raising further concerns about reproducibility.

The second major finding is its instability under permutation of feature order. In standard tabular analysis, the columns of a data matrix generally have no intrinsic order, and the direct tabular approach is invariant to such permutations. In contrast, once a tabular observation is serialized into text, the feature order becomes part of the input to the embedding model. Our results show that this arbitrary ordering can have a large impact on the resulting low-dimensional representation, in some cases reducing similarity to values close to zero. This is particularly concerning because the ordering information introduced during serialization is not intrinsic to the data itself, yet it can strongly influence the geometry of the embedding.

A natural question is why this same general strategy has shown promise in supervised learning problems, yet performs poorly for unsupervised dimension reduction. We believe the answer lies in the fundamental difference between supervised and unsupervised settings. In supervised learning, the outcome variable provides a clear target, and downstream model fitting can in effect filter out nuisance variation that is unhelpful for prediction. In unsupervised dimension reduction, by contrast, there is no such supervisory signal. The representation itself determines the geometry of the analysis. As a result, artificial variation introduced during serialization or embedding—such as sensitivity to missingness or feature order—can directly distort the low-dimensional structure.

At a more mechanistic level, the sensitivity of the LLM embedding-based approach to both missingness and feature order can be understood through the architecture of transformer models. The self-attention mechanism that underlies modern LLMs is, by itself, permutation-equivariant with respect to its input tokens (Vaswani et al., 2017). That is, without positional information, it treats its input as an unordered set. To enable the model to capture sequential structure, positional encodings are added to each token, making the model’s output dependent on the ordering of tokens in the input sequence. When tabular data are serialized into text, feature names and values become tokens, and their positions in the serialized string are encoded into the representation. Permuting the feature order therefore changes the positional encodings assigned to each token, which in turn can change the resulting embedding vector—even though the underlying tabular information is identical. This provides a concrete architectural explanation for the permutation sensitivity observed in our experiments.

A similar argument applies to missingness: when a feature value is present, the serialized text contains tokens for both the feature name and its numeric or categorical value, but when the value is missing, the value tokens are replaced by the word “missing.” Although the serialized text remains the same length in terms of feature–value pairs, the token-level content changes, which alters the attention patterns and can shift the resulting embedding of the entire observation. Even a small number of such substitutions can therefore propagate through the model in ways that are difficult to predict. It has also been noted more broadly that LLM-generated embeddings can be sensitive to small input perturbations (Tao et al., 2025), which is consistent with the instability we observe.

It is worth discussing the contexts in which stability of dimension reduction is important and, conversely, when some instability might be acceptable. Stability is critical whenever a low-dimensional representation is used as a primary analytical output from which scientific or clinical conclusions are drawn. This is common in exploratory data analysis, where researchers visually inspect low-dimensional plots to identify patient subgroups, discover disease subtypes, detect outliers, or assess data quality. If the resulting visualization changes substantially due to minor perturbations—such as a small amount of additional missingness or an arbitrary reordering of features—then the downstream biological or clinical interpretation becomes unreliable. Stability is also essential for reproducibility: ideally, two research groups analyzing the same dataset with the same pipeline should arrive at the same qualitative conclusions. In contrast, some instability may be more acceptable when dimension reduction serves only as an intermediate preprocessing step before a supervised task such as classification or regression. In that setting, the downstream labels provide a supervisory signal that can absorb moderate representational noise, and the final task performance—rather than the intermediate geometry—is the relevant criterion. However, this paper focuses specifically on the unsupervised setting, where the representation itself is the output and there is no such corrective signal. One might also ask whether instability under perturbation could serve as an informative diagnostic—for example, to identify influential features, analogous to influence diagnostics in regression. However, such an approach requires the method to be stable under changes that should have no effect, so that any observed instability can be attributed to a substantive modification. Since the LLM embedding-based approach is already unstable under mere permutation of feature order, which carries no information, one cannot reliably distinguish meaningful sensitivity from artifactual variation.

Taken together, our results indicate that the straightforward adaptation of LLM text embeddings from supervised tabular learning to unsupervised dimension reduction is problematic. This does not rule out the broader possibility that LLM-based representations may eventually be useful for dimension reduction of tabular data. Rather, it suggests that the current simple pipeline—serialize each row into text, embed the text, and directly use the resulting vectors for geometric analysis—is insufficiently stable for this purpose. Future work in this direction might consider representations that are by construction invariant to feature ordering and that degrade gracefully under missingness, rather than producing unpredictably different embeddings when the input is only slightly perturbed. More broadly, any embedding-based pipeline intended for unsupervised geometric analysis should be evaluated not only for downstream task performance but also for the stability of the representation itself.

## Data Availability

The source code for all analyses, including data preprocessing, embedding generation, stability evaluation, and figure generation, is available at https://github.com/jli-stat/llm-embedding-dimension-reduction-tabular.
