# Supplementary material for: Instability of LLM text embeddings for unsupervised dimension reduction of tabular data
Source: Front Bioinform. 2026 Jul 27;6:1851023. doi: 10.3389/fbinf.2026.1851023 (PMC13454057; doi:10.3389/fbinf.2026.1851023)
Supplement: Supplementary file 1 [file DataSheet1.pdf]

Supplementary Material

This supplement provides dataset descriptions, formal definitions of the distance measures used, and supplementary figures for robustness analyses.

1 Dataset Description and Data Preprocessing

1.1 Parkinson dataset

For the Oxford Parkinson's disease detection dataset (195 voice recordings from 31 individuals), we removed the status variable (0 = healthy, 1 = Parkinson's disease), which is the natural supervised-learning response in the original classification task. We also removed the name field because it is an identifier encoding subject and recording number rather than a biomedical measurement. All remaining columns correspond to real-valued dysphonia/voice measures (e.g., fundamental frequency statistics, jitter and shimmer descriptors, noise-to-harmonics ratios, and nonlinear dynamical features) and were retained as the unsupervised feature set. Values were coerced to numeric types, and any missing-value tokens encountered during import were standardized to NaN (the dataset documentation indicates no missing values).

1.2 WDBC dataset

For the Wisconsin Diagnostic Breast Cancer (WDBC) dataset (569 instances; 32 columns), we removed the ID number (an identifier that does not encode sample similarity) and the

diagnosis label (B/M), which is the natural response variable for supervised classification. The remaining 30 variables—corresponding to summary statistics (mean, standard error, and "worst") of 10 nucleus-level image features—were retained as the unsupervised feature set and were stored as numeric variables. Although the dataset documentation indicates no missing values, we still applied a standardized missingness parsing step during import (treating common missing tokens as missing and coercing any non-numeric entries to NaN) to ensure consistent handling across datasets.

1.3 HCV dataset

For the HCV dataset (hcvdat0.csv; patients with demographic variables and laboratory measurements), we removed the diagnosis label Category, which is the dataset's target attribute in supervised classification settings (blood donor status and hepatitis C disease stage). We also removed the patient identifier field (stored in this file as an ID/serial-number column) because it does not encode intrinsic patient similarity and can distort unsupervised embeddings. The remaining variables were retained as unsupervised features: Sex was treated as a nominal categorical variable, while Age and all laboratory measurements (e.g., ALB, ALP, ALT, AST, BIL, CHE, CHOL, CREA, GGT, PROT) were stored as numeric variables. Missing laboratory values (present in the source data) were parsed and represented as standard missing entries (NaN).

1.4 MIMIC-IV dataset

MIMIC-IV is a relational electronic health record database consisting of multiple tables describing patient demographics, hospital admissions, ICU stays, and clinical measurements. As in standard downstream analyses of clinical databases, we constructed a derived analytic table tailored to unsupervised dimension reduction, with one row per ICU stay. We restricted features to standard categorical and numerical variables that directly encode patient similarity without requiring task-specific feature engineering. Specifically, we included patient demographics (e.g., age, sex), admission characteristics (e.g., admission type, insurance, race), ICU stay descriptors (care unit and length of stay), and summary laboratory measurements. Laboratory variables were mapped using the MIMIC dictionary and aggregated within each ICU stay using simple statistics (mean, minimum, and maximum) to obtain a fixed-length

representation. Identifier variables (e.g., patient or encounter IDs) were excluded, as they do not encode meaningful similarity and can distort unsupervised embeddings. Absolute timestamp variables (e.g., admission or discharge times) were also excluded, since raw calendar time does not reflect intrinsic clinical similarity without additional modeling assumptions. This resulted in a clinically interpretable, moderate-dimensional tabular dataset representative of those commonly visualized using unsupervised dimension reduction methods in biomedical research. All experiments using MIMIC-IV were conducted on the publicly available demo subset.

1.5 eICU dataset

The eICU Collaborative Research Database is a multi-center critical care database containing de-identified electronic health records from intensive care units across the United States. As in standard downstream analyses of clinical databases, we constructed a derived analytic table tailored to unsupervised dimension reduction, with one row per ICU stay. We restricted features to standard categorical and numerical variables that directly encode patient similarity without requiring task-specific feature engineering. Specifically, we included patient demographics (e.g., age, sex, ethnicity), ICU unit and administrative descriptors (e.g., ICU type, admission source, discharge status), anthropometric measurements (height and weight), severity-of-illness summaries derived from APACHE IVa, and summary laboratory measurements. When multiple APACHE versions were available for the same ICU stay, we retained APACHE IVa to ensure a single severity representation per stay. Laboratory variables were aggregated within each ICU stay using simple statistics (mean, minimum, and maximum) to obtain a fixed-length representation without temporal modeling. Identifier variables (e.g., patient or encounter identifiers) were excluded, as they do not encode meaningful similarity and can distort unsupervised embeddings. Absolute timestamp variables and time offsets were also excluded, since raw calendar time does not reflect intrinsic clinical similarity without additional modeling assumptions. This resulted in a clinically interpretable, moderate-dimensional tabular dataset representative of those commonly visualized using unsupervised dimension reduction methods in biomedical research. All experiments using eICU were conducted on the publicly available demo subset.

1.6 NHANES dataset

NHANES is a cross-sectional, population-based health survey containing demographic, socioeconomic, behavioral, and clinical measurement variables in a single flat table. For unsupervised dimension reduction, we used the curated NHANES dataset provided by the NHANES R package and retained the standard categorical and numerical variables describing participant characteristics, health behaviors, and measured biomarkers. We excluded non-informative variables, including participant identifiers and survey administration indicators, as well as redundant encodings of the same construct (e.g., discretized age variables, repeated blood pressure measurements, and derived BMI categories). When multiple representations were available, we retained the most informative summary or continuous form (e.g., averaged blood pressure, continuous BMI, numeric household income midpoint). We also removed child-specific screen-time variables to avoid mixing age-specific semantics. This resulted in a heterogeneous tabular dataset with substantial real-world missingness, suitable for evaluating the stability of unsupervised dimension reduction methods.

1.7 Hepatitis dataset

For the Hepatitis dataset (155 instances; 20 attributes including a class label), we removed the class attribute (DIE/LIVE), which serves as the natural response variable in supervised settings, to enforce a fully unsupervised analysis. All remaining 19 attributes were retained as candidate features. Variables encoded as binary clinical indicators (e.g., steroid use, antivirals, fatigue, anorexia, hepatomegaly, ascites, varices, and histology) were treated as nominal categorical variables and recoded from the original 1,2 representation to no, yes; sex was similarly treated as nominal and recoded from 1,2 to male, female. Age and laboratory measurements (bilirubin, alkaline phosphatase, SGOT, albumin, and prothrombin time) were retained as numeric variables. Missing values, indicated by “?” in the raw file, were parsed and stored as standard missing entries (NaN) in the resulting DataFrame.

1.8 Thyroid-diff dataset

For the Differentiated Thyroid Cancer Recurrence dataset (clinicopathologic variables collected over a 15-year period with long-term follow-up), we removed Recurred, the dataset's target variable indicating whether recurrence occurred, to enforce a fully unsupervised setting. No identifier or timestamp fields were present in the provided CSV file, so no additional non-feature columns were removed. Age was retained as a numeric feature. All remaining clinicopathologic variables (e.g., gender, smoking history, thyroid function, physical examination, adenopathy, pathology, focality, risk, TNM components, stage, and response) were treated as nominal categorical features and stored using pandas' categorical dtype; we did not impose an ordinal structure because explicit, dataset-provided level orderings were not required for the downstream unsupervised stability analysis. Although the repository documentation indicates no missing values, we still standardized missingness during import by mapping common missing tokens to NaN to ensure consistent preprocessing across datasets.

2 Gower distance for mixed-type data

Consider a dataset with n observations and p features. Let x_{ik} denote the value of feature k for observation i. The Gower distance between two observations i and j is defined as a weighted average of feature-wise dissimilarities:

$$D_{ij} = \frac{\sum_{k=1}^{p} w_k \, \delta_{ijk} \, d_{ijk}}{\sum_{k=1}^{p} w_k \, \delta_{ijk}}, \qquad i \neq j,$$

where $w_k \geq 0$ is the weight of feature k, $d_{ijk} \in [0, 1]$ is the dissimilarity for feature k, and $\delta_{ijk} \in \{0, 1\}$ indicates whether feature k is comparable for the pair (i, j).

In general, $\delta_{ijk} = 1$ if both x_{ik} and x_{jk} are observed, and $\delta_{ijk} = 0$ otherwise. Thus, the distance is computed using only the features observed for both observations (pairwise deletion).

The feature-wise dissimilarity d_{ijk} depends on the feature type:

- **Continuous features:**
$$d_{ijk} = \frac{|x_{ik} - x_{jk}|}{R_k},$$
where R_k is the range of feature k (e.g., $\max x_{\cdot k} - \min x_{\cdot k}$, or a robust quantile-based range).

- **Ordinal features:**
$$d_{ijk} = \frac{|r_{ik} - r_{jk}|}{m_k - 1},$$
where r_{ik} is the rank (or ordered category code) and m_k is the number of levels.

- **Nominal features:**
$$d_{ijk} = \mathbf{1}\{x_{ik} \neq x_{jk}\}.$$

- **Symmetric binary features:**
$$d_{ijk} = \mathbf{1}\{x_{ik} \neq x_{jk}\}.$$

- **Asymmetric binary features:** Let $z_{ik} = \mathbf{1}\{x_{ik} = 1\}$ denote presence. Then
$$d_{ijk} = \mathbf{1}\{z_{ik} \neq z_{jk}\},$$
and $\delta_{ijk} = 0$ when $(z_{ik}, z_{jk}) = (0, 0)$, so joint absences are ignored.

If the denominator $\sum_k w_k \delta_{ijk}$ is zero (i.e., no comparable features), we set $D_{ij} = 1$. The diagonal entries are defined as $D_{ii} = 0$.

All feature-wise dissimilarities are scaled to lie in $[0, 1]$, so that $D_{ij} \in [0, 1]$ and is directly comparable across mixed feature types.

3 NaN-aware Euclidean distance

To define a Euclidean-type distance for mixed-type tabular data with missing values, we first transform all features into a common numeric representation.

3.1 Feature transformation

Each feature is mapped to one or more numeric variables as follows:

- **Continuous features:**
$$z_{ik} = \frac{x_{ik} - \min x_{\cdot k}}{\max x_{\cdot k} - \min x_{\cdot k}},$$
so that values are scaled to $[0, 1]$.

- **Ordinal features:**
$$z_{ik} = \frac{r_{ik}}{m_k - 1},$$
where r_{ik} is the rank (or category code) and m_k is the number of levels. If ordinal structure is not explicitly available, a range-based scaling is used as for continuous variables.

- **Binary features:** Values are encoded as $z_{ik} \in \{0, 1\}$.

- **Nominal features:** Each nominal feature is expanded via one-hot encoding. If feature k has L_k categories, it is replaced by L_k binary variables.

Missing values are preserved throughout the transformation: if x_{ik} is missing, the corresponding transformed entries are set to missing.

Let $\mathbf{z}_i \in \mathbb{R}^M$ denote the resulting expanded numeric vector for observation i, where M is the total number of transformed dimensions.

3.2 Distance definition

For two observations i and j, define the set of comparable dimensions

$$\mathcal{K}_{ij} = \{k : z_{ik} \text{ and } z_{jk} \text{ are both observed}\},$$

and let $m_{ij} = |\mathcal{K}_{ij}|$.

The NaN-aware Euclidean distance is defined as

$$D_{ij} = \sqrt{\frac{M}{m_{ij}} \sum_{k \in \mathcal{K}_{ij}} (z_{ik} - z_{jk})^2}, \qquad i \neq j.$$

Thus, the squared differences are computed only over comparable dimensions, and then rescaled by M/m_{ij} to account for missingness.

3.3 Special cases

If $m_{ij} = 0$, i.e., the two observations share no comparable dimensions, we set

$$D_{ij} = 1.$$

The diagonal entries are defined as $D_{ii} = 0$.

3.4 Remarks

All transformed features lie in $[0, 1]$, so the distance aggregates contributions from mixed feature types in a comparable manner. The rescaling factor M/m_{ij} ensures that distances remain on a comparable scale even when the number of observed dimensions varies across pairs.

4 HEOM distance

We also consider the Heterogeneous Euclidean-Overlap Metric (HEOM), which defines a distance for mixed-type data by combining feature-wise dissimilarities.

4.1 Feature-wise dissimilarities

For each feature k, a dissimilarity $d_{ijk} \in [0, 1]$ is defined as follows:

- **Continuous features:**

$$d_{ijk} = \frac{|x_{ik} - x_{jk}|}{R_k},$$

  where $R_k = \max x_{\cdot k} - \min x_{\cdot k}$.

- **Ordinal features:**

$$d_{ijk} = \frac{|r_{ik} - r_{jk}|}{m_k - 1},$$

  where r_{ik} is the rank (or category code) and m_k is the number of levels. A range-based scaling is used if ordinal structure is not explicitly available.

- **Nominal and binary features:**

$$d_{ijk} = \mathbf{1}\{x_{ik} \neq x_{jk}\}.$$

4.2 Handling of missing values

If either x_{ik} or x_{jk} is missing, the feature contributes maximal dissimilarity:

$$d_{ijk} = 1.$$

If a feature contains no observed values across all samples, it is excluded from the computation.

4.3 Distance definition

Let p' denote the number of features used in the computation (excluding entirely missing features). The HEOM distance is defined as

$$D_{ij} = \sqrt{\frac{1}{p'} \sum_{k=1}^{p'} d_{ijk}^2}, \qquad i \neq j.$$

The diagonal entries are defined as $D_{ii} = 0$.

4.4 Remarks

Each feature contributes equally to the distance, and all feature-wise dissimilarities are bounded in $[0, 1]$. Unlike the Gower distance, HEOM treats missing values as maximally dissimilar rather than omitting them, which may increase sensitivity to missingness.

Supplementary Tables

Table S1: Summary of variables included in the derived eICU-CRD ICU-stay analytic table. Identifier variables and absolute timestamp variables were excluded prior to dimension reduction.

Variable	Type	Source	Description
Demographic variables			
gender	Categorical	patient	Patient sex
age	Numeric	patient	Patient age (years; top-coded in eICU)
ethnicity	Categorical	patient	Patient-reported ethnicity
ICU unit and administrative descriptors			
unittype	Categorical	patient	ICU type (e.g., Med-Surg ICU, SICU)
unitadmitsource	Categorical	patient	Source of ICU admission
unitstaytype	Categorical	patient	Type of ICU stay (e.g., admit, stepdown)
hospitaldischargestatus	Categorical	patient	Hospital discharge status (e.g., Alive, Expired)
unitdischargestatus	Categorical	patient	ICU discharge status (e.g., Alive, Expired)
Anthropometric variables			
admissionheight	Numeric	patient	Height at admission (cm)
admissionweight	Numeric	patient	Weight at admission (kg)
dischargeweight	Numeric	patient	Weight at ICU discharge (kg)
Severity of illness and outcome summaries (APACHE IVa)			
acutephysiologyscore	Numeric	apachePatientResult	Acute Physiology Score component of APACHE
apachescore	Numeric	apachePatientResult	Overall APACHE score
predictedicumortality	Numeric	apachePatientResult	Predicted ICU mortality probability

Continued on next page

Variable	Type	Source	Description
actualicumortality	Categorical	apachePatientResult	Observed ICU mortality outcome
predictediculos	Numeric	apachePatientResult	Predicted ICU length of stay (days)
actualiculos	Numeric	apachePatientResult	Observed ICU length of stay (days)
predictedhospitalmortality	Numeric	apachePatientResult	Predicted hospital mortality probability
actualhospitalmortality	Categorical	apachePatientResult	Observed hospital mortality outcome
predictedhospitallos	Numeric	apachePatientResult	Predicted hospital length of stay (days)
actualhospitallos	Numeric	apachePatientResult	Observed hospital length of stay (days)
actualventdays	Numeric	apachePatientResult	Observed duration of mechanical ventilation (days)
predventdays	Numeric	apachePatientResult	Predicted duration of mechanical ventilation (days)
Laboratory summary features (aggregated within ICU stay)			
mean_hgb	Numeric	lab	Mean hemoglobin level during ICU stay
min_hgb	Numeric	lab	Minimum hemoglobin level during ICU stay
max_hgb	Numeric	lab	Maximum hemoglobin level during ICU stay
mean_hct	Numeric	lab	Mean hematocrit during ICU stay
min_hct	Numeric	lab	Minimum hematocrit during ICU stay
max_hct	Numeric	lab	Maximum hematocrit during ICU stay

Continued on next page

Variable	Type	Source	Description
mean_wbc_x_1000	Numeric	lab	Mean white blood cell count during ICU stay
min_wbc_x_1000	Numeric	lab	Minimum white blood cell count during ICU stay
max_wbc_x_1000	Numeric	lab	Maximum white blood cell count during ICU stay
mean_platelets_x_1000	Numeric	lab	Mean platelet count during ICU stay
min_platelets_x_1000	Numeric	lab	Minimum platelet count during ICU stay
max_platelets_x_1000	Numeric	lab	Maximum platelet count during ICU stay
mean_rbc	Numeric	lab	Mean red blood cell count during ICU stay
min_rbc	Numeric	lab	Minimum red blood cell count during ICU stay
max_rbc	Numeric	lab	Maximum red blood cell count during ICU stay
mean_sodium	Numeric	lab	Mean serum sodium level during ICU stay
min_sodium	Numeric	lab	Minimum serum sodium level during ICU stay
max_sodium	Numeric	lab	Maximum serum sodium level during ICU stay
mean_potassium	Numeric	lab	Mean serum potassium level during ICU stay
min_potassium	Numeric	lab	Minimum serum potassium level during ICU stay
max_potassium	Numeric	lab	Maximum serum potassium level during ICU stay

Continued on next page

Variable	Type	Source	Description
mean_chloride	Numeric	lab	Mean serum chloride level during ICU stay
min_chloride	Numeric	lab	Minimum serum chloride level during ICU stay
max_chloride	Numeric	lab	Maximum serum chloride level during ICU stay
mean_co2	Numeric	lab	Mean serum bicarbonate (CO_2) during ICU stay
min_co2	Numeric	lab	Minimum serum bicarbonate (CO_2) during ICU stay
max_co2	Numeric	lab	Maximum serum bicarbonate (CO_2) during ICU stay
mean_creatinine	Numeric	lab	Mean serum creatinine level during ICU stay
min_creatinine	Numeric	lab	Minimum serum creatinine level during ICU stay
max_creatinine	Numeric	lab	Maximum serum creatinine level during ICU stay
mean_bun	Numeric	lab	Mean blood urea nitrogen (BUN) during ICU stay
min_bun	Numeric	lab	Minimum blood urea nitrogen (BUN) during ICU stay
max_bun	Numeric	lab	Maximum blood urea nitrogen (BUN) during ICU stay
mean_glucose	Numeric	lab	Mean serum glucose level during ICU stay
min_glucose	Numeric	lab	Minimum serum glucose level during ICU stay
max_glucose	Numeric	lab	Maximum serum glucose level during ICU stay

Continued on next page

Variable	Type	Source	Description
mean_lactate	Numeric	lab	Mean serum lactate level during ICU stay
min_lactate	Numeric	lab	Minimum serum lactate level during ICU stay
max_lactate	Numeric	lab	Maximum serum lactate level during ICU stay

Table S2: Summary of variables included in the derived MIMIC-IV ICU-stay analytic table. Identifier variables and absolute timestamps were excluded prior to dimension reduction.

**Variable**	**Type**	**Source**	**Description**
Demographic variables			
gender	Categorical	patients	Patient sex
anchor_age	Numeric	patients	Patient age (de-identified anchor age)
Admission-level characteristics			
admission_type	Categorical	admissions	Type of hospital admission (e.g., emergency, elective, urgent)
admission_location	Categorical	admissions	Location prior to hospital admission
insurance	Categorical	admissions	Primary insurance type
language	Categorical	admissions	Primary language (if recorded)
marital_status	Categorical	admissions	Marital status (if recorded)
race	Categorical	admissions	Race (as recorded in MIMIC-IV)
hospital_expire_flag	Binary	admissions	Indicator of in-hospital mortality
ICU stay descriptors			
first_careunit	Categorical	icustays	Initial ICU care unit during the stay
last_careunit	Categorical	icustays	Final ICU care unit during the stay
los	Numeric	icustays	ICU length of stay (days)
Laboratory summary features (aggregated within ICU stay)			
mean_creatinine	Numeric	labevents	Mean serum creatinine level during ICU stay
min_creatinine	Numeric	labevents	Minimum serum creatinine level during ICU stay

Continued on next page

Variable	Type	Source	Description
max_creatinine	Numeric	labevents	Maximum serum creatinine level during ICU stay
mean_urea_nitrogen	Numeric	labevents	Mean blood urea nitrogen (BUN) during ICU stay
min_urea_nitrogen	Numeric	labevents	Minimum blood urea nitrogen during ICU stay
max_urea_nitrogen	Numeric	labevents	Maximum blood urea nitrogen during ICU stay
mean_sodium	Numeric	labevents	Mean serum sodium level during ICU stay
min_sodium	Numeric	labevents	Minimum serum sodium level during ICU stay
max_sodium	Numeric	labevents	Maximum serum sodium level during ICU stay
mean_potassium	Numeric	labevents	Mean serum potassium level during ICU stay
min_potassium	Numeric	labevents	Minimum serum potassium level during ICU stay
max_potassium	Numeric	labevents	Maximum serum potassium level during ICU stay
mean_chloride	Numeric	labevents	Mean serum chloride level during ICU stay
min_chloride	Numeric	labevents	Minimum serum chloride level during ICU stay
max_chloride	Numeric	labevents	Maximum serum chloride level during ICU stay
mean_bicarbonate	Numeric	labevents	Mean serum bicarbonate level during ICU stay
min_bicarbonate	Numeric	labevents	Minimum serum bicarbonate level during ICU stay

Continued on next page

Variable	Type	Source	Description
max_bicarbonate	Numeric	labevents	Maximum serum bicarbonate level during ICU stay
mean_glucose	Numeric	labevents	Mean serum glucose level during ICU stay
min_glucose	Numeric	labevents	Minimum serum glucose level during ICU stay
max_glucose	Numeric	labevents	Maximum serum glucose level during ICU stay
mean_hemoglobin	Numeric	labevents	Mean hemoglobin concentration during ICU stay
min_hemoglobin	Numeric	labevents	Minimum hemoglobin concentration during ICU stay
max_hemoglobin	Numeric	labevents	Maximum hemoglobin concentration during ICU stay
mean_white_blood_cells	Numeric	labevents	Mean white blood cell count during ICU stay
min_white_blood_cells	Numeric	labevents	Minimum white blood cell count during ICU stay
max_white_blood_cells	Numeric	labevents	Maximum white blood cell count during ICU stay
mean_platelet_count	Numeric	labevents	Mean platelet count during ICU stay
min_platelet_count	Numeric	labevents	Minimum platelet count during ICU stay
max_platelet_count	Numeric	labevents	Maximum platelet count during ICU stay
mean_lactate	Numeric	labevents	Mean serum lactate level during ICU stay
min_lactate	Numeric	labevents	Minimum serum lactate level during ICU stay

Continued on next page

Variable	Type	Source	Description
max_lactate	Numeric	labevents	Maximum serum lactate level during ICU stay
mean_albumin	Numeric	labevents	Mean serum albumin level during ICU stay
min_albumin	Numeric	labevents	Minimum serum albumin level during ICU stay
max_albumin	Numeric	labevents	Maximum serum albumin level during ICU stay
mean_bilirubin_total	Numeric	labevents	Mean total bilirubin level during ICU stay
min_bilirubin_total	Numeric	labevents	Minimum total bilirubin level during ICU stay
max_bilirubin_total	Numeric	labevents	Maximum total bilirubin level during ICU stay
mean_anion_gap	Numeric	labevents	Mean anion gap during ICU stay
min_anion_gap	Numeric	labevents	Minimum anion gap during ICU stay
max_anion_gap	Numeric	labevents	Maximum anion gap during ICU stay

Supplementary Figures

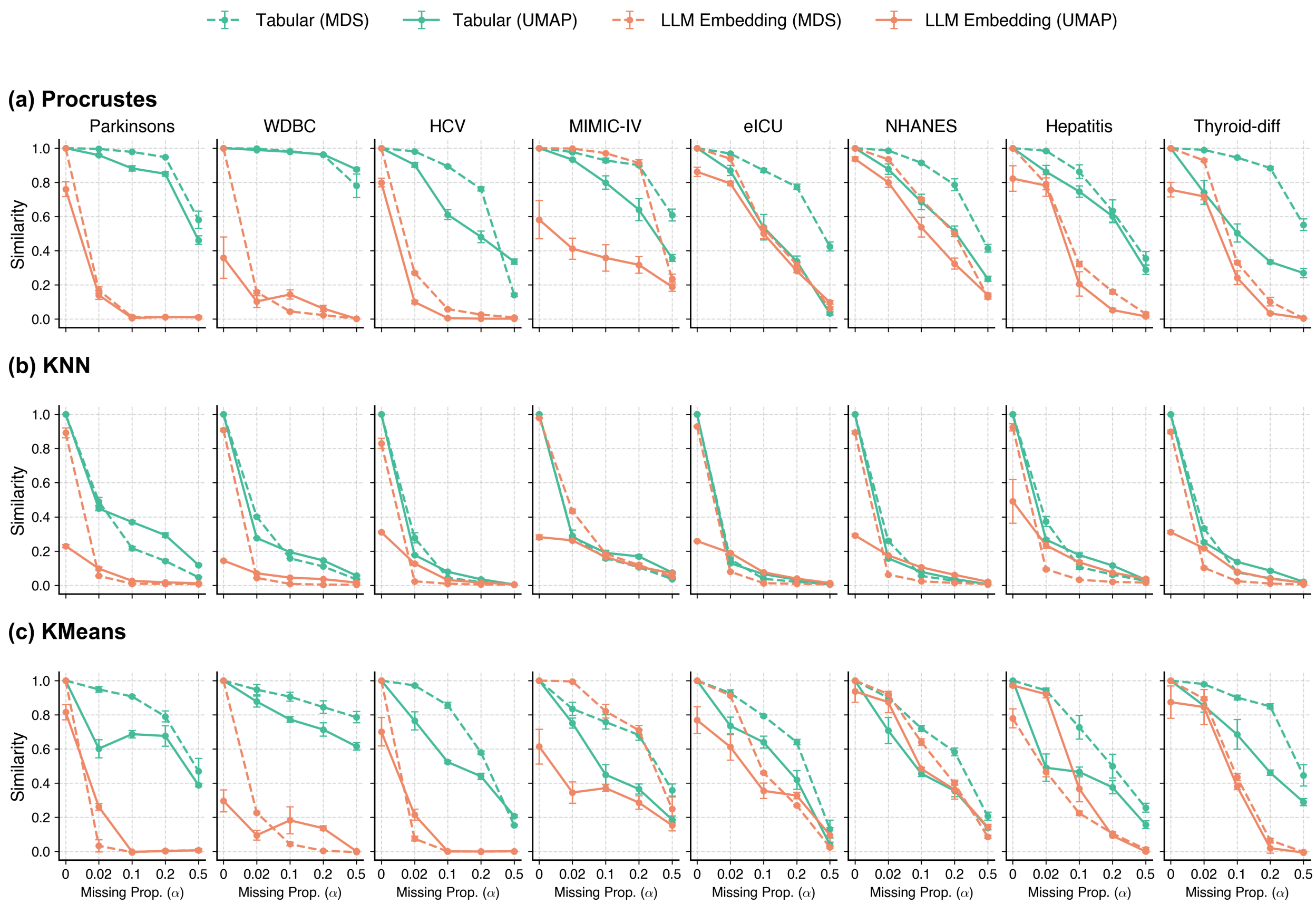

Figure S1: **Dimension reduction stability under missing completely at random (MCAR) when Euclidean distance is used for the embedding-based approach.** Additional missing values were introduced independently into each cell with probability α. Stability was assessed by comparing the resulting low-dimensional representation with that obtained from the original data using three complementary measures: (a) Procrustes similarity, (b) KNN similarity, and (c) KMeans similarity. Higher values indicate greater stability, with 1 corresponding to perfect agreement and values near 0 indicating little meaningful agreement. For the LLM embedding-based approach, pairwise distances between embeddings were computed using Euclidean distance; results for the direct tabular approach are shown for comparison. Both approaches were combined with either MDS or UMAP.

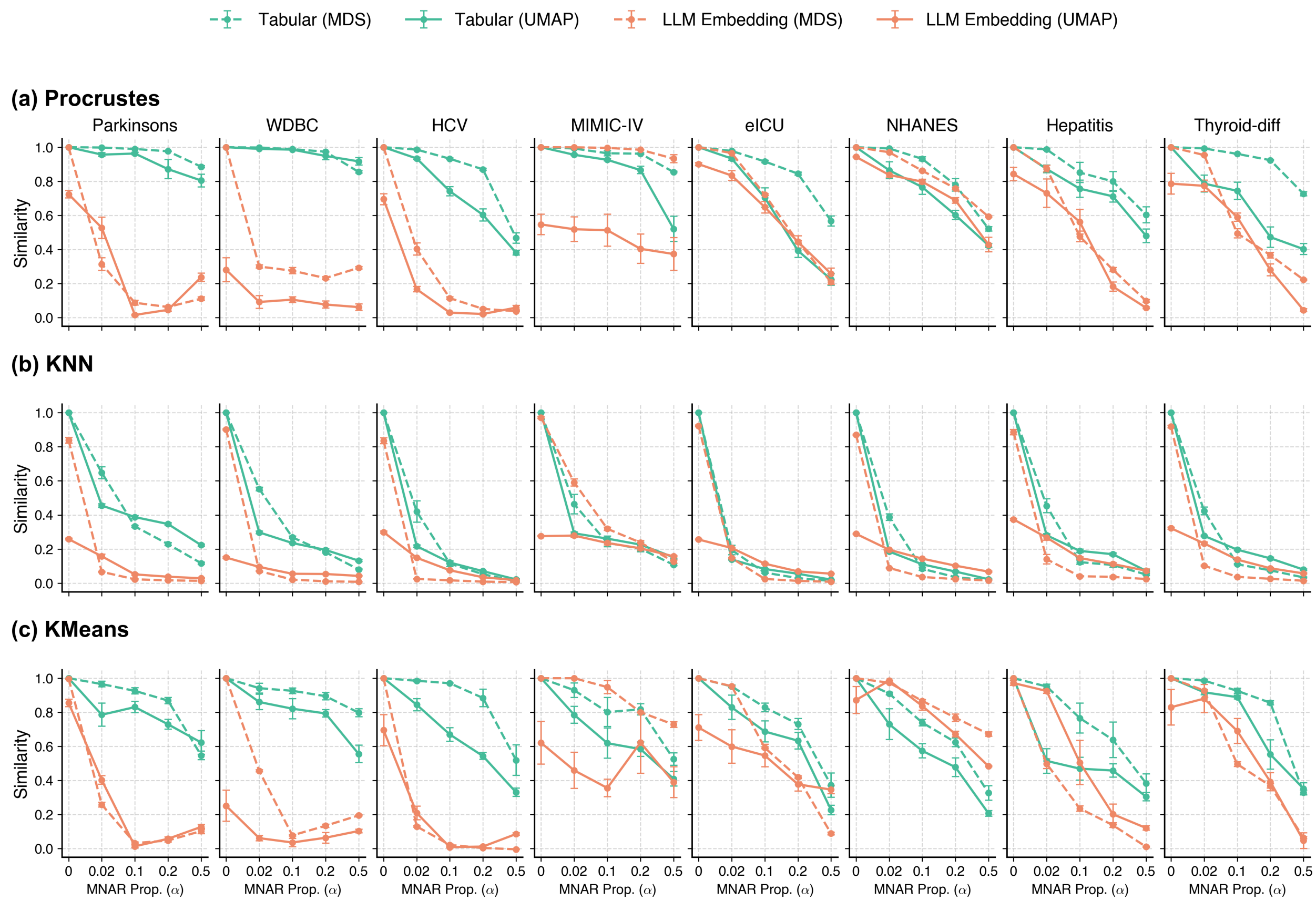

Figure S2: **Dimension reduction stability under missing not at random (MNAR) when Euclidean distance is used for the embedding-based approach.** Additional missing values were introduced using a structured missingness mechanism, and the resulting low-dimensional representation was compared with that from the original data. Stability was quantified using (a) Procrustes similarity, (b) KNN similarity, and (c) KMeans similarity. Higher values indicate greater stability, with 1 corresponding to perfect agreement and values near 0 indicating little meaningful agreement. For the LLM embedding-based approach, pairwise distances between embeddings were computed using Euclidean distance; results for the direct tabular approach are shown for comparison. Both approaches were combined with either MDS or UMAP.

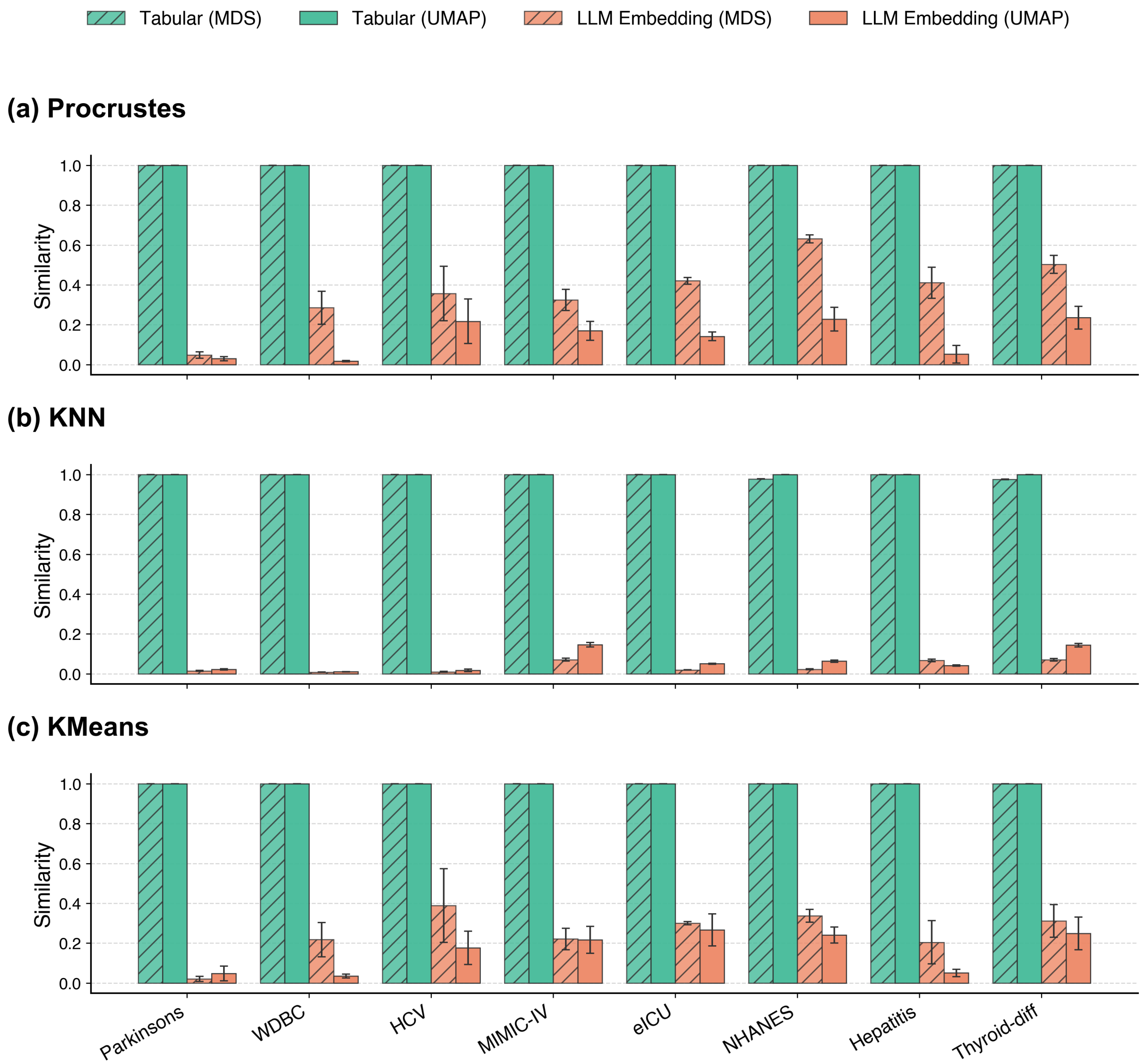

Figure S3: **Dimension reduction stability under permutation of feature order when Euclidean distance is used for the embedding-based approach.** For each dataset, the columns of the tabular data were randomly permuted, and the resulting low-dimensional representation was compared with that obtained from the original unpermuted data. Stability was quantified using (a) Procrustes similarity, (b) KNN similarity, and (c) KMeans similarity. Higher values indicate greater stability, with 1 corresponding to perfect agreement and values near 0 indicating little meaningful agreement. For the LLM embedding-based approach, pairwise distances between embeddings were computed using Euclidean distance; results for the direct tabular approach are shown for comparison. Both approaches were combined with either MDS or UMAP.

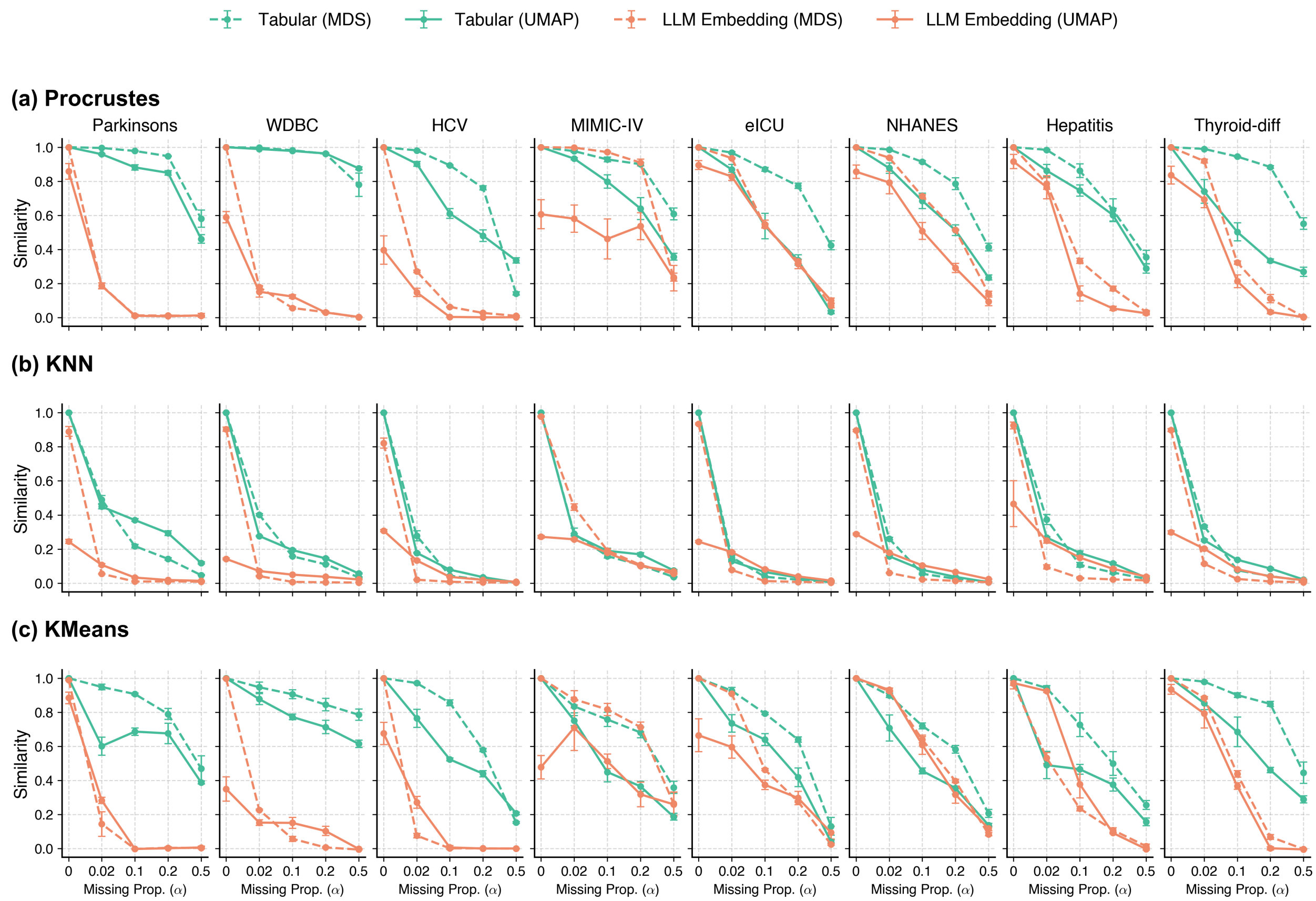

Figure S4: **Dimension reduction stability under missing completely at random (MCAR) when standardized Euclidean distance is used for the embedding-based approach.** Additional missing values were introduced independently into each cell with probability α. Stability was assessed by comparing the resulting low-dimensional representation with that obtained from the original data using three complementary measures: (a) Procrustes similarity, (b) KNN similarity, and (c) KMeans similarity. Higher values indicate greater stability, with 1 corresponding to perfect agreement and values near 0 indicating little meaningful agreement. For the LLM embedding-based approach, pairwise distances between embeddings were computed using Euclidean distance after standardizing each embedding dimension; results for the direct tabular approach are shown for comparison. Both approaches were combined with either MDS or UMAP.

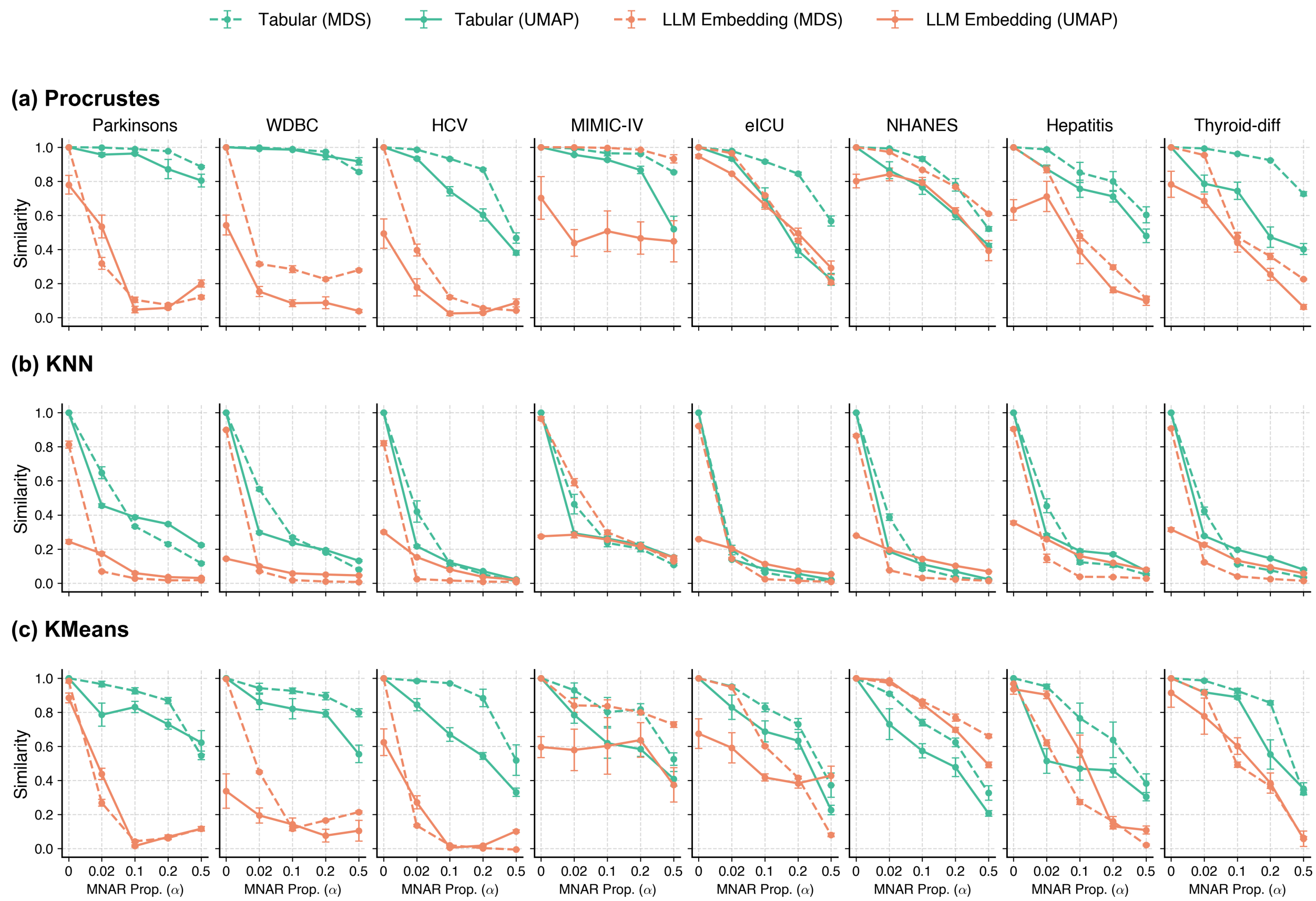

Figure S5: **Dimension reduction stability under missing not at random (MNAR) when standardized Euclidean distance is used for the embedding-based approach.** Additional missing values were introduced using a structured missingness mechanism, and the resulting low-dimensional representation was compared with that from the original data. Stability was quantified using (a) Procrustes similarity, (b) KNN similarity, and (c) KMeans similarity. Higher values indicate greater stability, with 1 corresponding to perfect agreement and values near 0 indicating little meaningful agreement. For the LLM embedding-based approach, pairwise distances between embeddings were computed using Euclidean distance after standardizing each embedding dimension; results for the direct tabular approach are shown for comparison. Both approaches were combined with either MDS or UMAP.

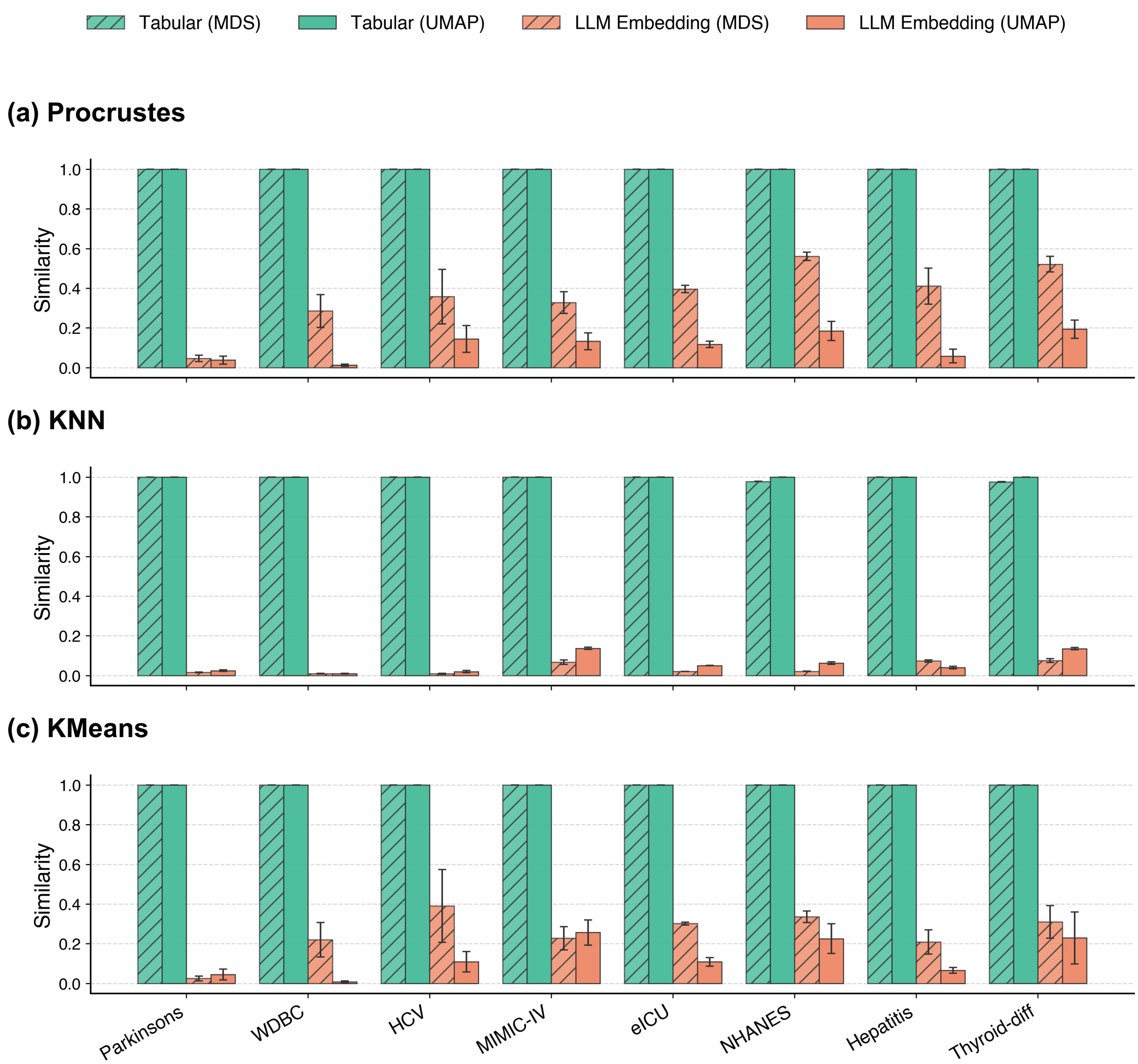

Figure S6: **Dimension reduction stability under permutation of feature order when standardized Euclidean distance is used for the embedding-based approach.** For each dataset, the columns of the tabular data were randomly permuted, and the resulting low-dimensional representation was compared with that obtained from the original unpermuted data. Stability was quantified using (a) Procrustes similarity, (b) KNN similarity, and (c) KMeans similarity. Higher values indicate greater stability, with 1 corresponding to perfect agreement and values near 0 indicating little meaningful agreement. For the LLM embedding-based approach, pairwise distances between embeddings were computed using Euclidean distance after standardizing each embedding dimension; results for the direct tabular approach are shown for comparison. Both approaches were combined with either MDS or UMAP.

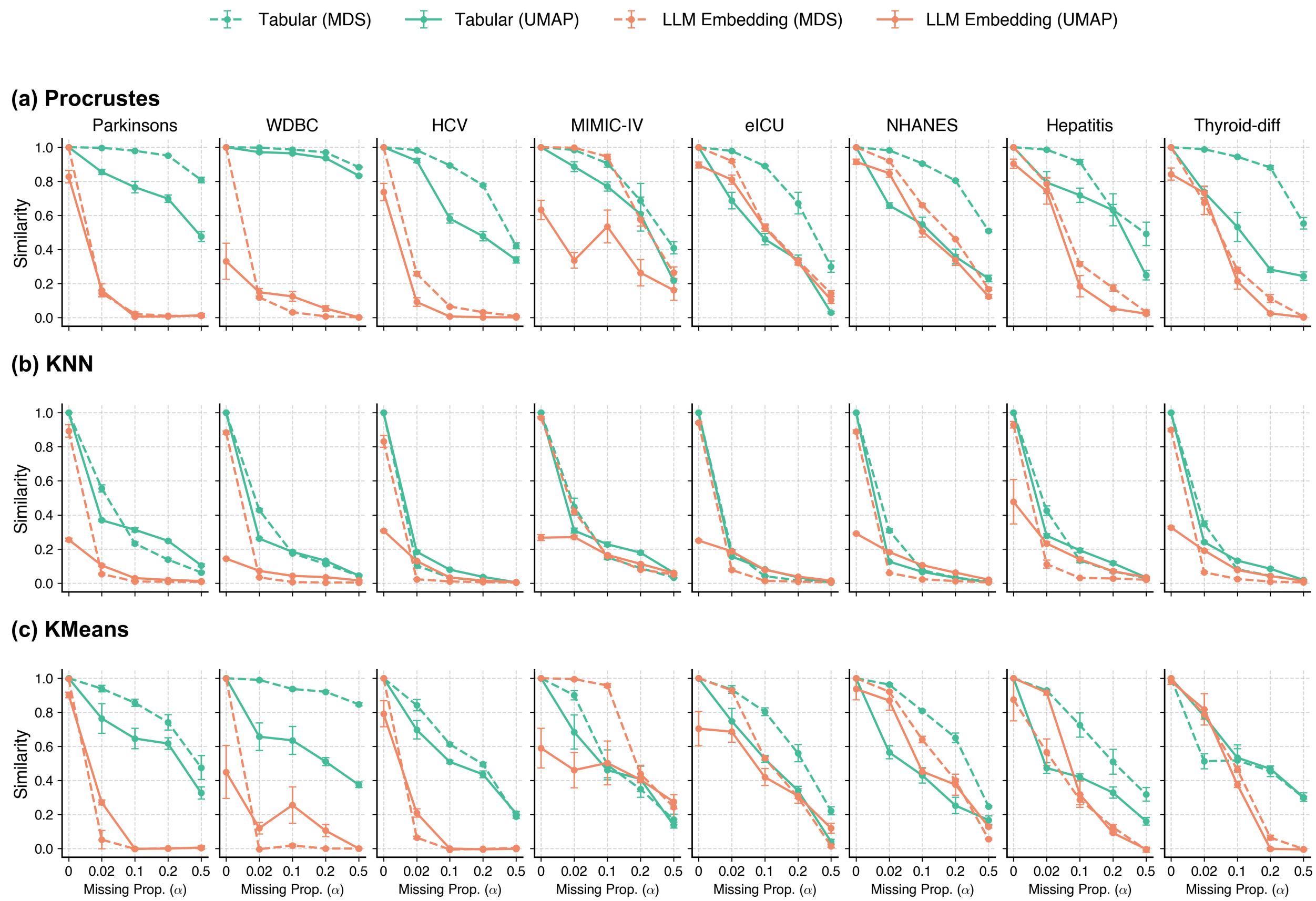

Figure S7: **Dimension reduction stability under missing completely at random (MCAR) when the direct tabular approach uses the NaN-aware Euclidean distance.** Additional missing values were introduced independently into each cell with probability α. Stability was assessed by comparing the resulting low-dimensional representation with that obtained from the original data using three complementary measures: (a) Procrustes similarity, (b) KNN similarity, and (c) KMeans similarity. Higher values indicate greater stability, with 1 corresponding to perfect agreement and values near 0 indicating little meaningful agreement. For the direct tabular approach, pairwise distances were computed using the NaN-aware Euclidean distance; results for the LLM embedding-based approach are shown for comparison. Both approaches were combined with either MDS or UMAP.

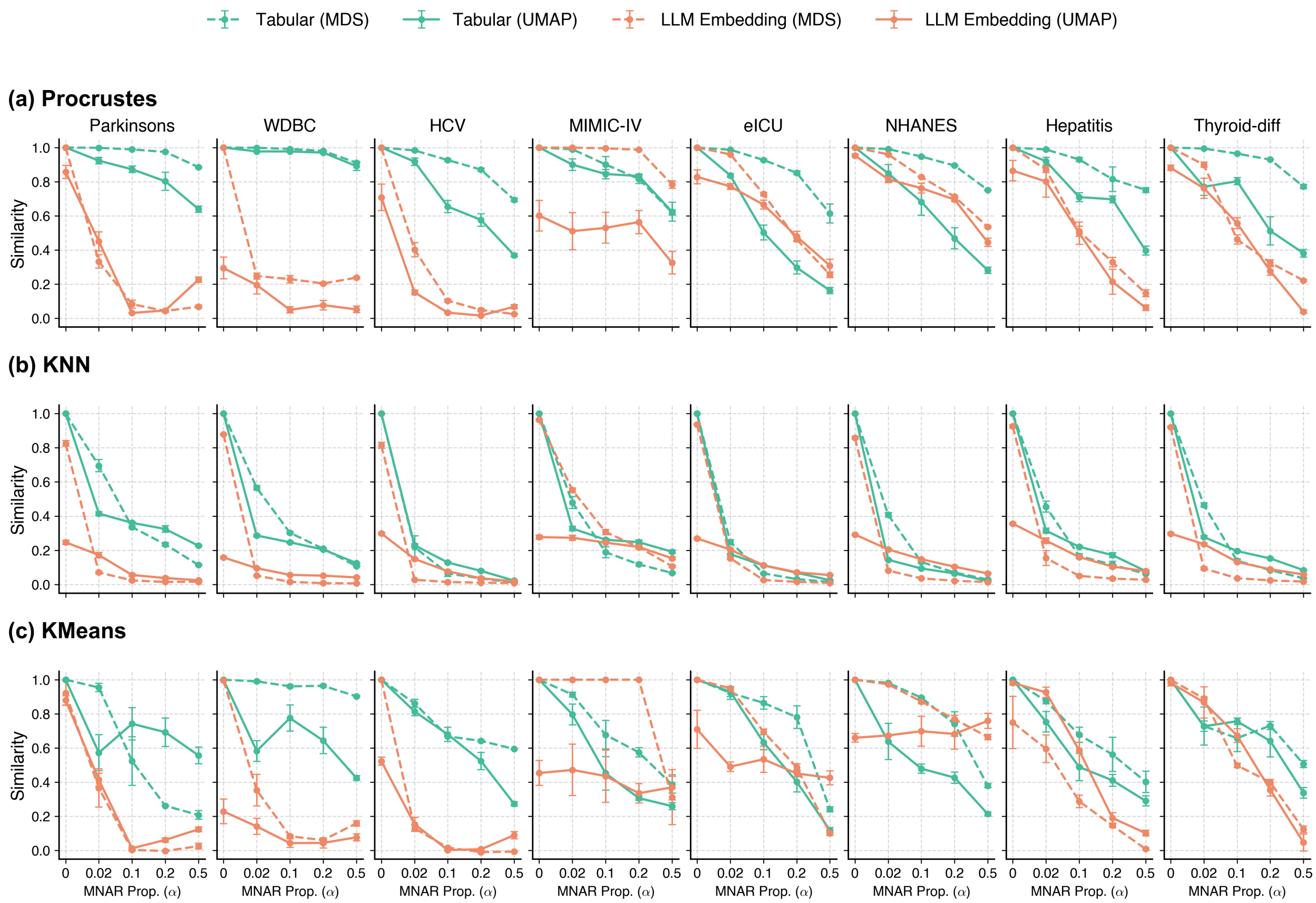

Figure S8: **Dimension reduction stability under missing not at random (MNAR) when the direct tabular approach uses the NaN-aware Euclidean distance.** Additional missing values were introduced using a structured missingness mechanism, and the resulting low-dimensional representation was compared with that from the original data. Stability was quantified using (a) Procrustes similarity, (b) KNN similarity, and (c) KMeans similarity. Higher values indicate greater stability, with 1 corresponding to perfect agreement and values near 0 indicating little meaningful agreement. For the direct tabular approach, pairwise distances were computed using the NaN-aware Euclidean distance; results for the LLM embedding-based approach are shown for comparison. Both approaches were combined with either MDS or UMAP.

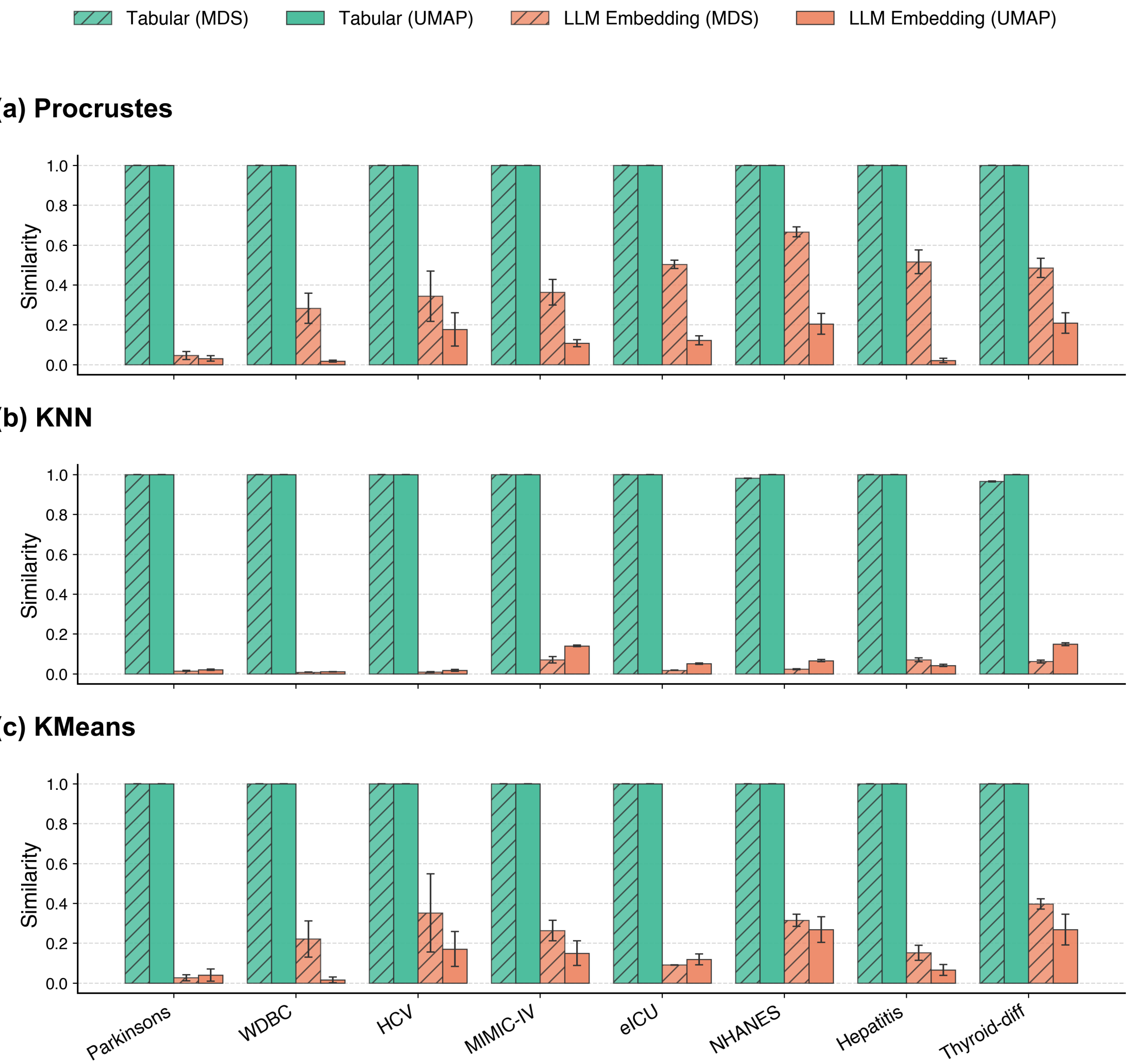

Figure S9: **Dimension reduction stability under permutation of feature order when the direct tabular approach uses the NaN-aware Euclidean distance.** For each dataset, the columns of the tabular data were randomly permuted, and the resulting low-dimensional representation was compared with that obtained from the original unpermuted data. Stability was quantified using (a) Procrustes similarity, (b) KNN similarity, and (c) KMeans similarity. Higher values indicate greater stability, with 1 corresponding to perfect agreement and values near 0 indicating little meaningful agreement. For the direct tabular approach, pairwise distances were computed using the NaN-aware Euclidean distance; results for the LLM embedding-based approach are shown for comparison. Both approaches were combined with either MDS or UMAP.

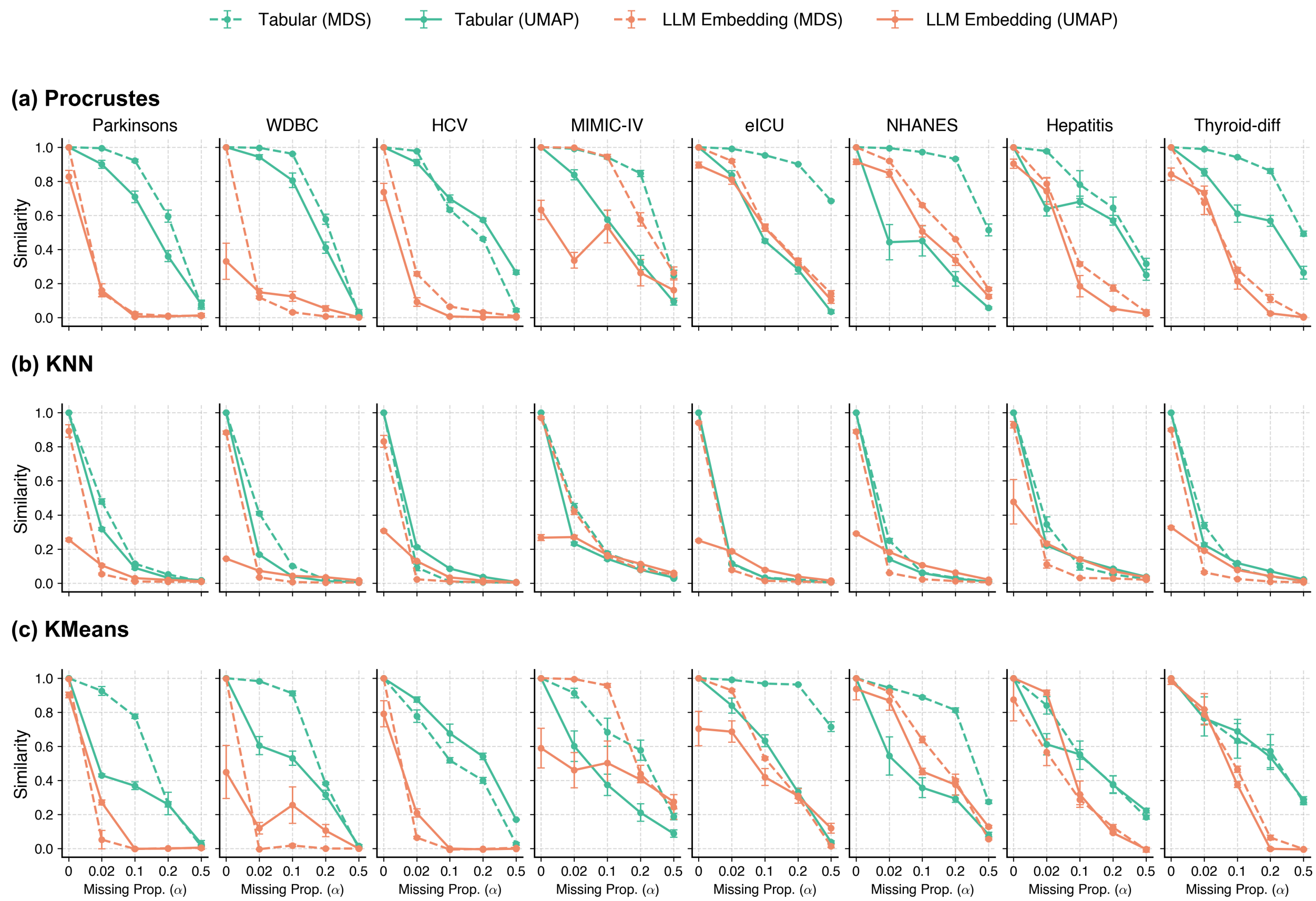

Figure S10: **Dimension reduction stability under missing completely at random (MCAR) when the direct tabular approach uses HEOM.** Additional missing values were introduced independently into each cell with probability α. Stability was assessed by comparing the resulting low-dimensional representation with that obtained from the original data using three complementary measures: (a) Procrustes similarity, (b) KNN similarity, and (c) KMeans similarity. Higher values indicate greater stability, with 1 corresponding to perfect agreement and values near 0 indicating little meaningful agreement. For the direct tabular approach, pairwise distances were computed using the Heterogeneous Euclidean-Overlap Metric (HEOM); results for the LLM embedding-based approach are shown for comparison. Both approaches were combined with either MDS or UMAP.

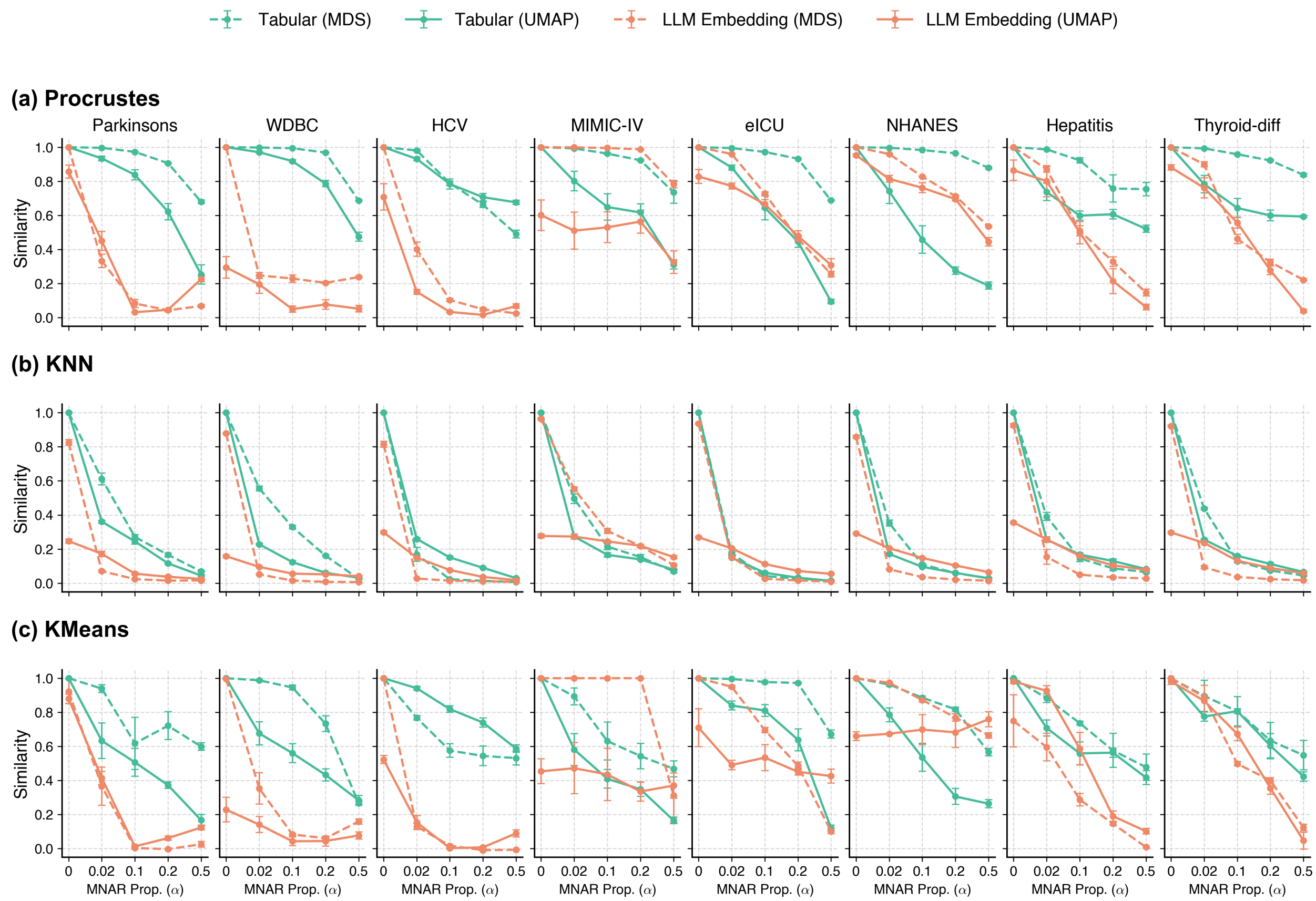

Figure S11: **Dimension reduction stability under missing not at random (MNAR) when the direct tabular approach uses HEOM.** Additional missing values were introduced using a structured missingness mechanism, and the resulting low-dimensional representation was compared with that from the original data. Stability was quantified using (a) Procrustes similarity, (b) KNN similarity, and (c) KMeans similarity. Higher values indicate greater stability, with 1 corresponding to perfect agreement and values near 0 indicating little meaningful agreement. For the direct tabular approach, pairwise distances were computed using the Heterogeneous Euclidean-Overlap Metric (HEOM); results for the LLM embedding-based approach are shown for comparison. Both approaches were combined with either MDS or UMAP.

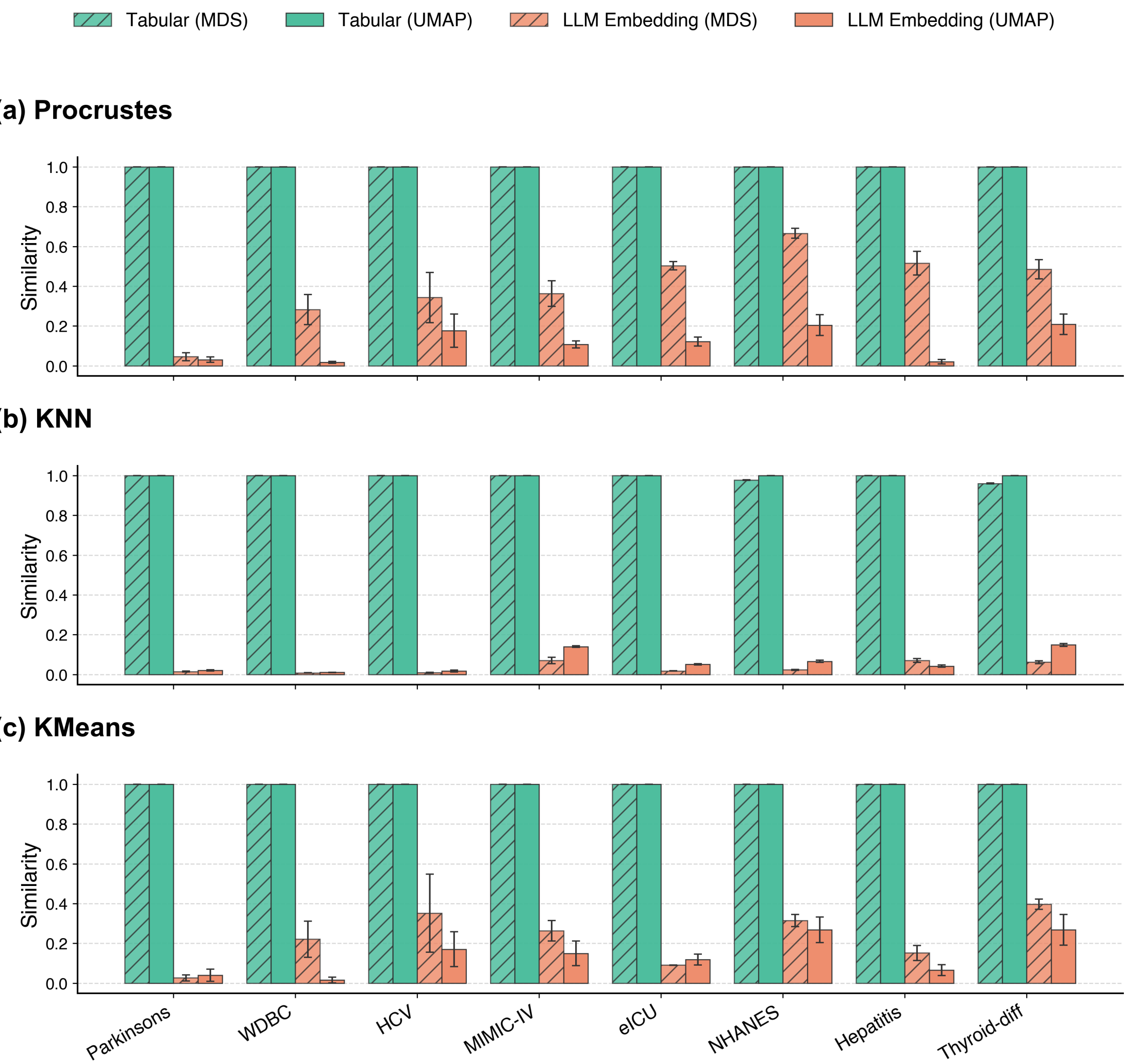

Figure S12: **Dimension reduction stability under permutation of feature order when the direct tabular approach uses HEOM.** For each dataset, the columns of the tabular data were randomly permuted, and the resulting low-dimensional representation was compared with that obtained from the original unpermuted data. Stability was quantified using (a) Procrustes similarity, (b) KNN similarity, and (c) KMeans similarity. Higher values indicate greater stability, with 1 corresponding to perfect agreement and values near 0 indicating little meaningful agreement. For the direct tabular approach, pairwise distances were computed using the Heterogeneous Euclidean-Overlap Metric (HEOM); results for the LLM embedding-based approach are shown for comparison. Both approaches were combined with either MDS or UMAP.

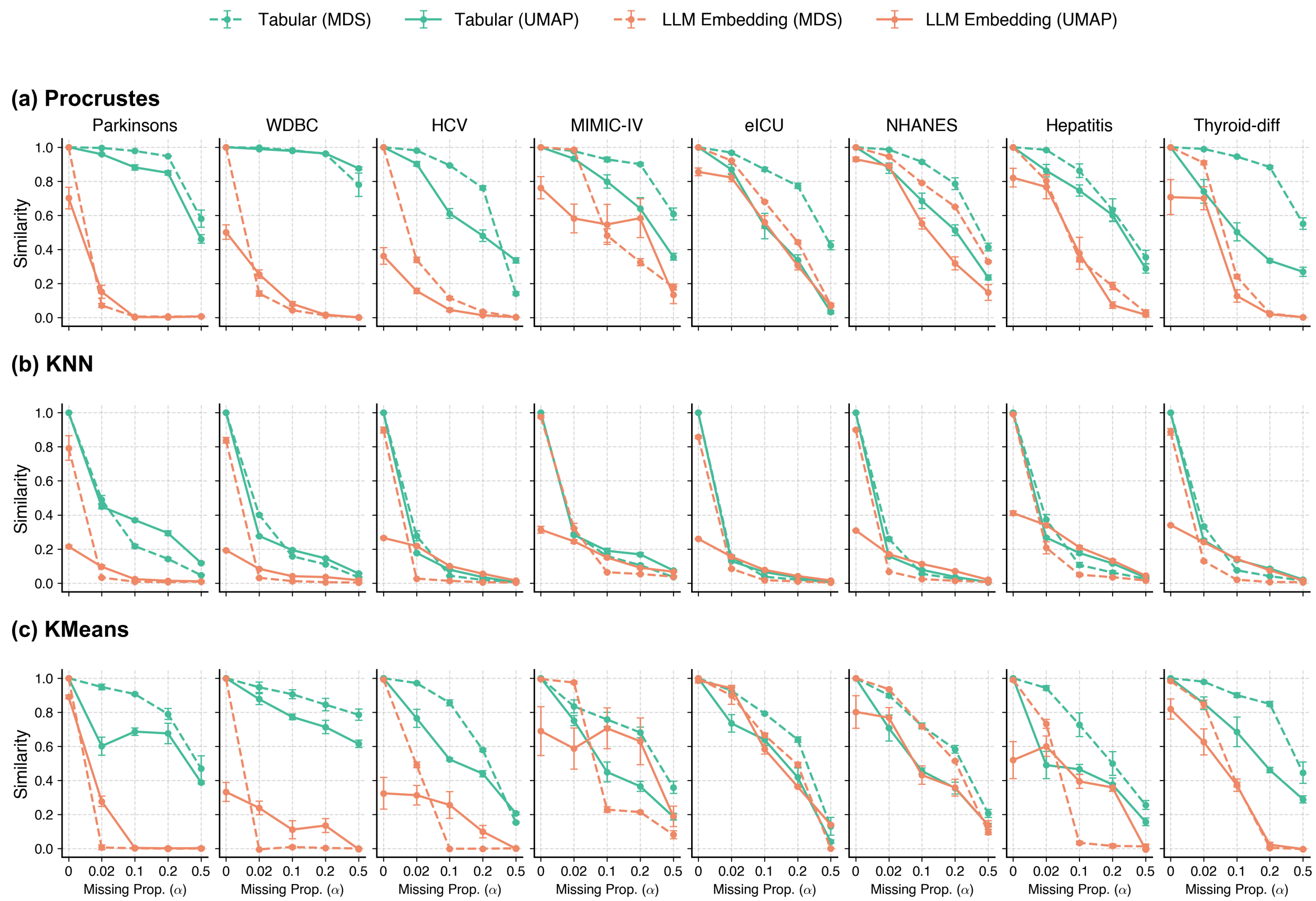

Figure S13: **Dimension reduction stability under missing completely at random (MCAR) when the "is" template is used for the embedding-based approach.** Additional missing values were introduced independently into each cell with probability α. Stability was assessed by comparing the resulting low-dimensional representation with that obtained from the original data using three complementary measures: (a) Procrustes similarity, (b) KNN similarity, and (c) KMeans similarity. Higher values indicate greater stability, with 1 corresponding to perfect agreement and values near 0 indicating little meaningful agreement. For the LLM embedding-based approach, each observation was serialized using a template of the form "Feature is value" before computing embeddings; results for the direct tabular approach are shown for comparison. Both approaches were combined with either MDS or UMAP.

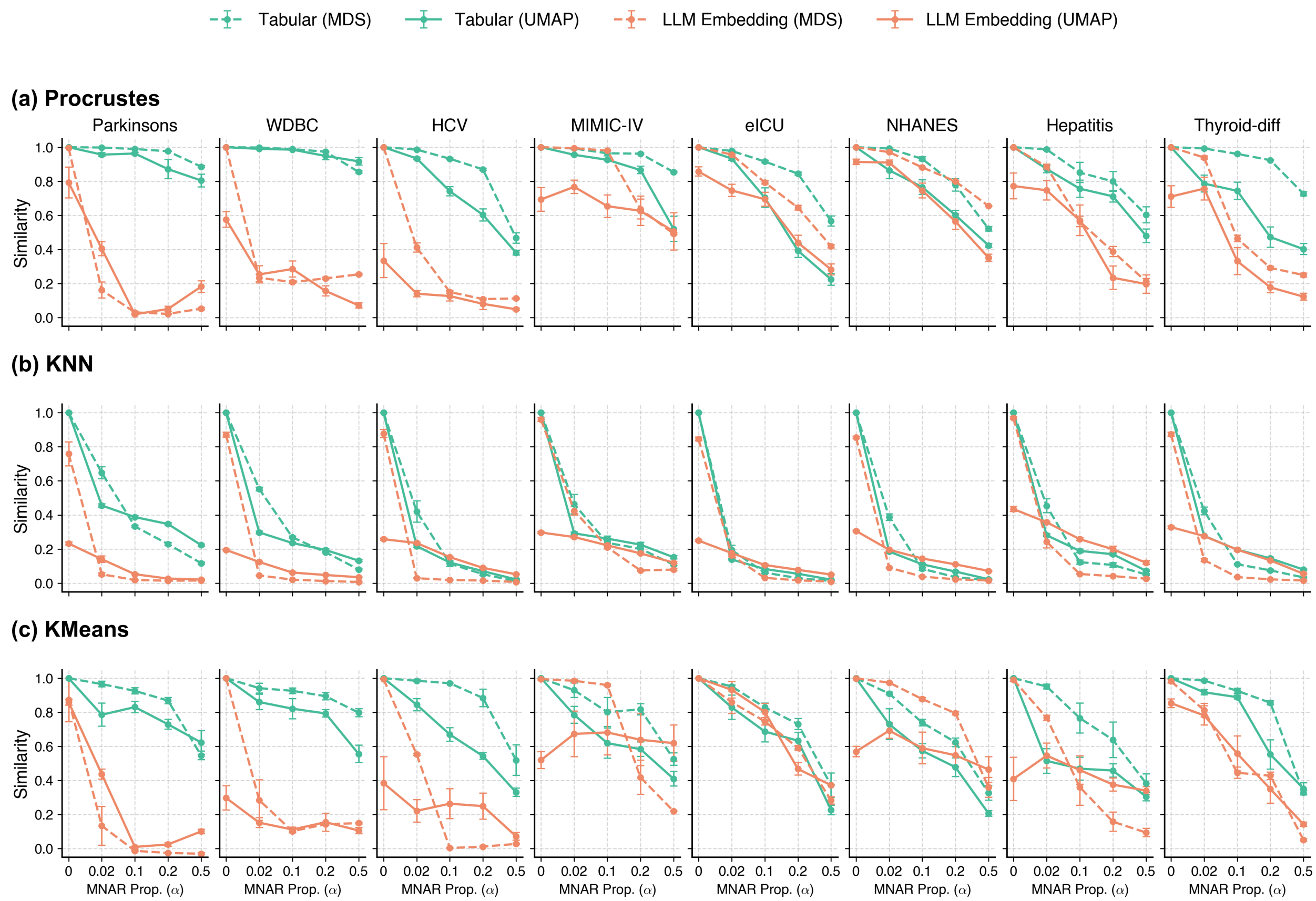

Figure S14: **Dimension reduction stability under missing not at random (MNAR) when the "is" template is used for the embedding-based approach.** Additional missing values were introduced using a structured missingness mechanism, and the resulting low-dimensional representation was compared with that from the original data. Stability was quantified using (a) Procrustes similarity, (b) KNN similarity, and (c) KMeans similarity. Higher values indicate greater stability, with 1 corresponding to perfect agreement and values near 0 indicating little meaningful agreement. For the LLM embedding-based approach, each observation was serialized using a template of the form "Feature is value" before computing embeddings; results for the direct tabular approach are shown for comparison. Both approaches were combined with either MDS or UMAP.

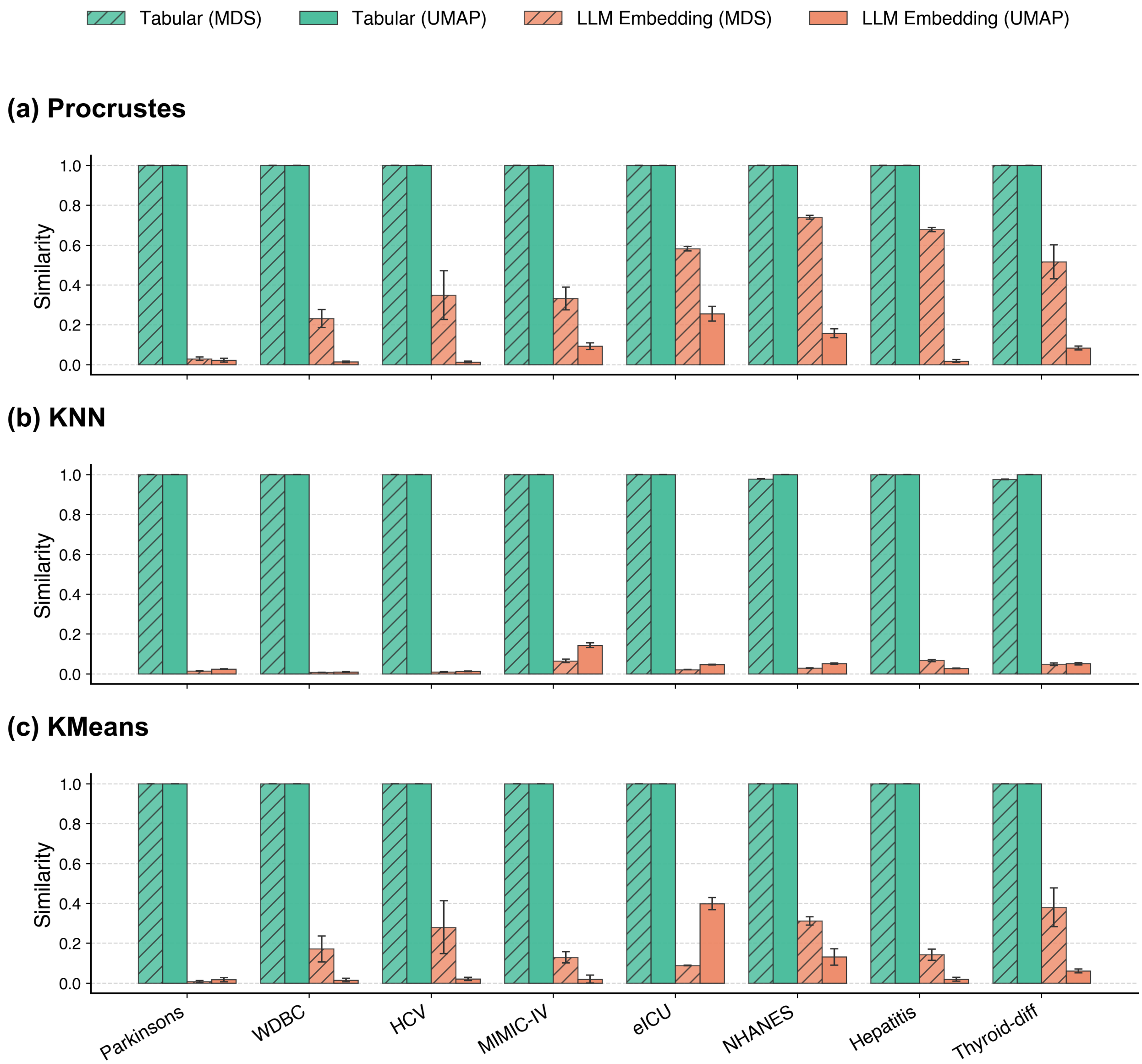

Figure S15: **Dimension reduction stability under permutation of feature order when the "is" template is used for the embedding-based approach.** For each dataset, the columns of the tabular data were randomly permuted, and the resulting low-dimensional representation was compared with that obtained from the original unpermuted data. Stability was quantified using (a) Procrustes similarity, (b) KNN similarity, and (c) KMeans similarity. Higher values indicate greater stability, with 1 corresponding to perfect agreement and values near 0 indicating little meaningful agreement. For the LLM embedding-based approach, each observation was serialized using a template of the form "Feature is value" before computing embeddings; results for the direct tabular approach are shown for comparison. Both approaches were combined with either MDS or UMAP.

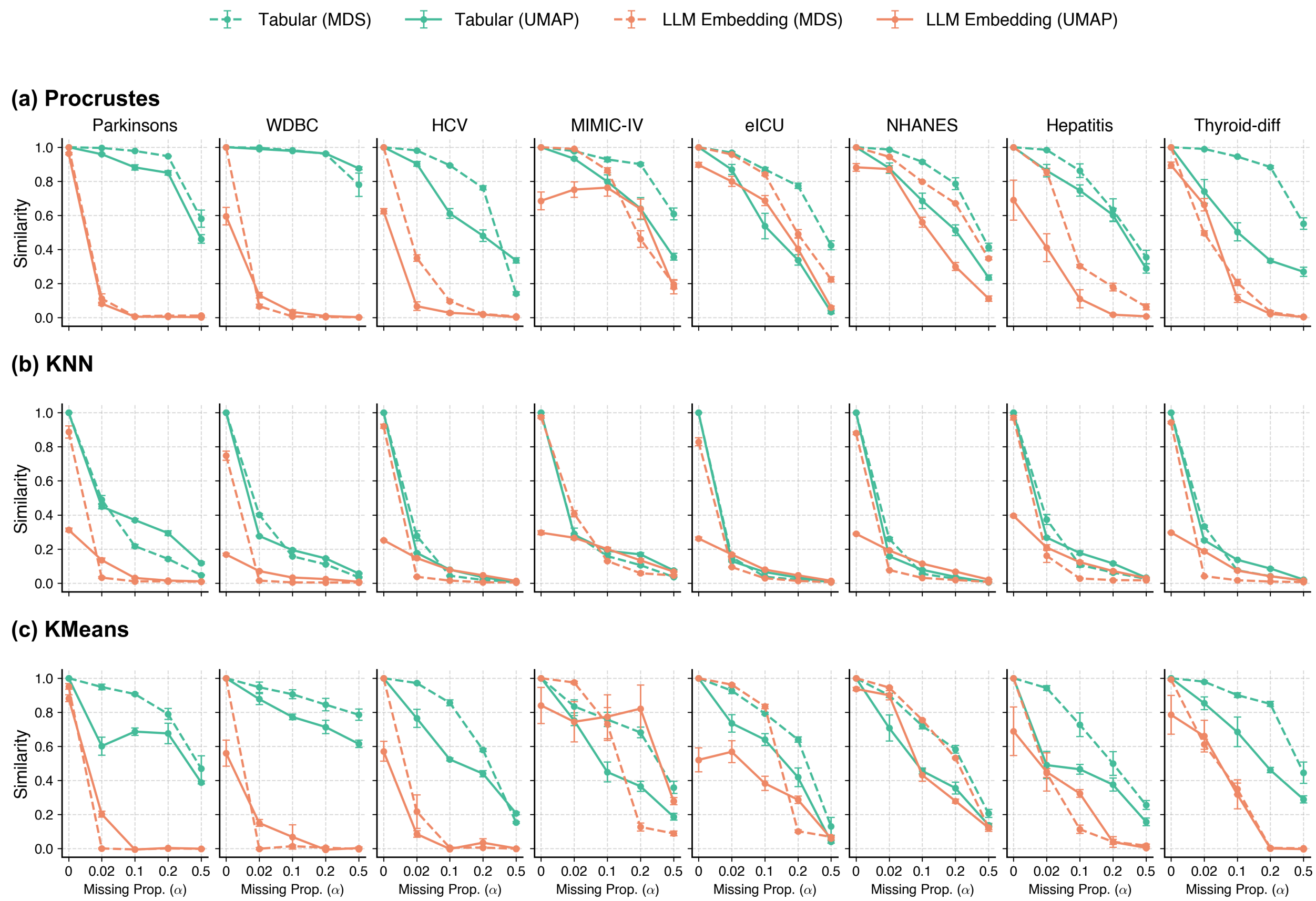

Figure S16: **Dimension reduction stability under missing completely at random (MCAR) when a JSON serialization template is used for the embedding-based approach.** Additional missing values were introduced independently into each cell with probability α. Stability was assessed by comparing the resulting low-dimensional representation with that obtained from the original data using three complementary measures: (a) Procrustes similarity, (b) KNN similarity, and (c) KMeans similarity. Higher values indicate greater stability, with 1 corresponding to perfect agreement and values near 0 indicating little meaningful agreement. For the LLM embedding-based approach, each observation was serialized using a JSON format (e.g., `{"Age": 27, "Gender": "F"}`) before computing embeddings; results for the direct tabular approach are shown for comparison. Both approaches were combined with either MDS or UMAP.

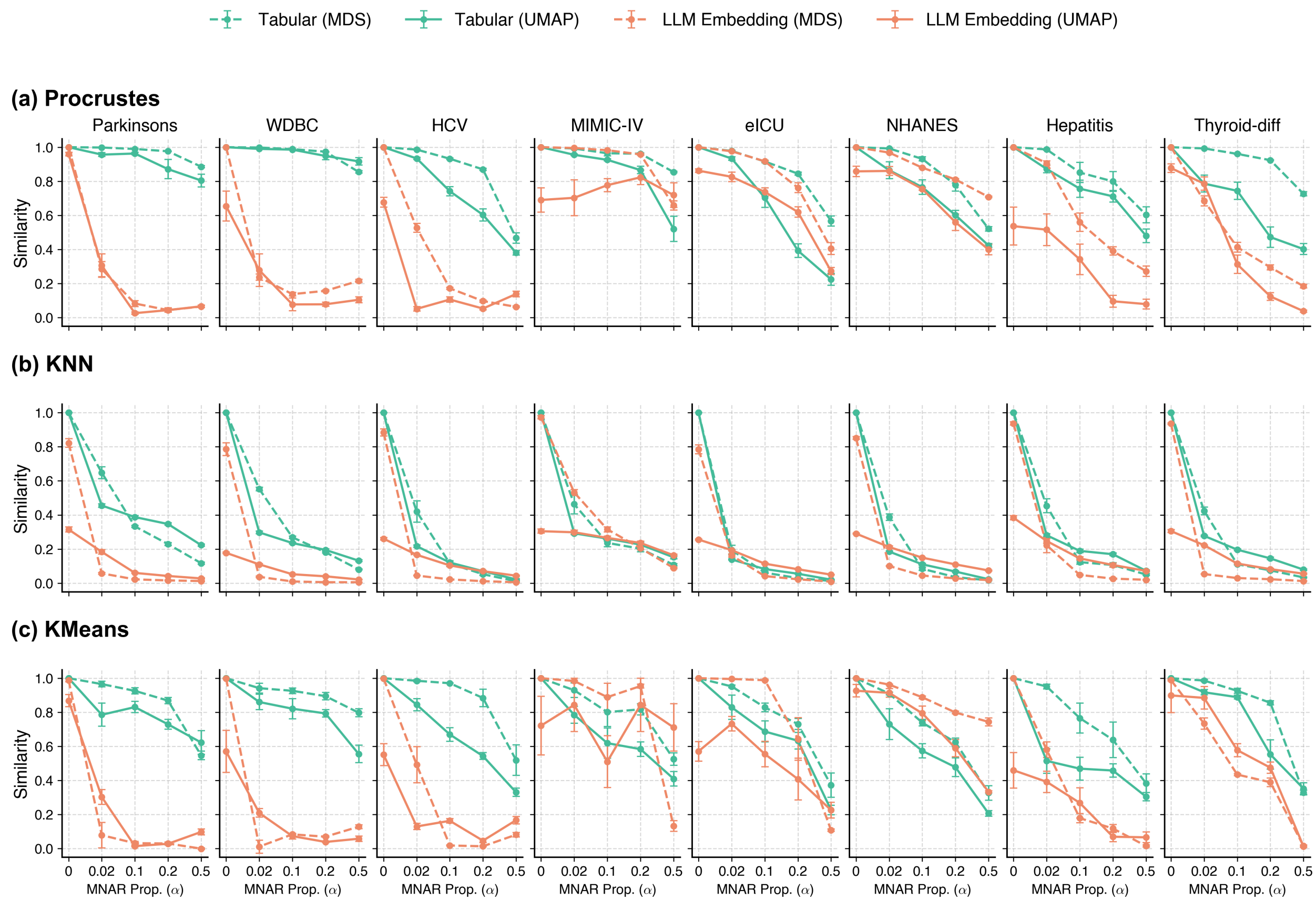

Figure S17: **Dimension reduction stability under missing not at random (MNAR) when a JSON serialization template is used for the embedding-based approach.** Additional missing values were introduced using a structured missingness mechanism, and the resulting low-dimensional representation was compared with that from the original data. Stability was quantified using (a) Procrustes similarity, (b) KNN similarity, and (c) KMeans similarity. Higher values indicate greater stability, with 1 corresponding to perfect agreement and values near 0 indicating little meaningful agreement. For the LLM embedding-based approach, each observation was serialized using a JSON format before computing embeddings; results for the direct tabular approach are shown for comparison. Both approaches were combined with either MDS or UMAP.

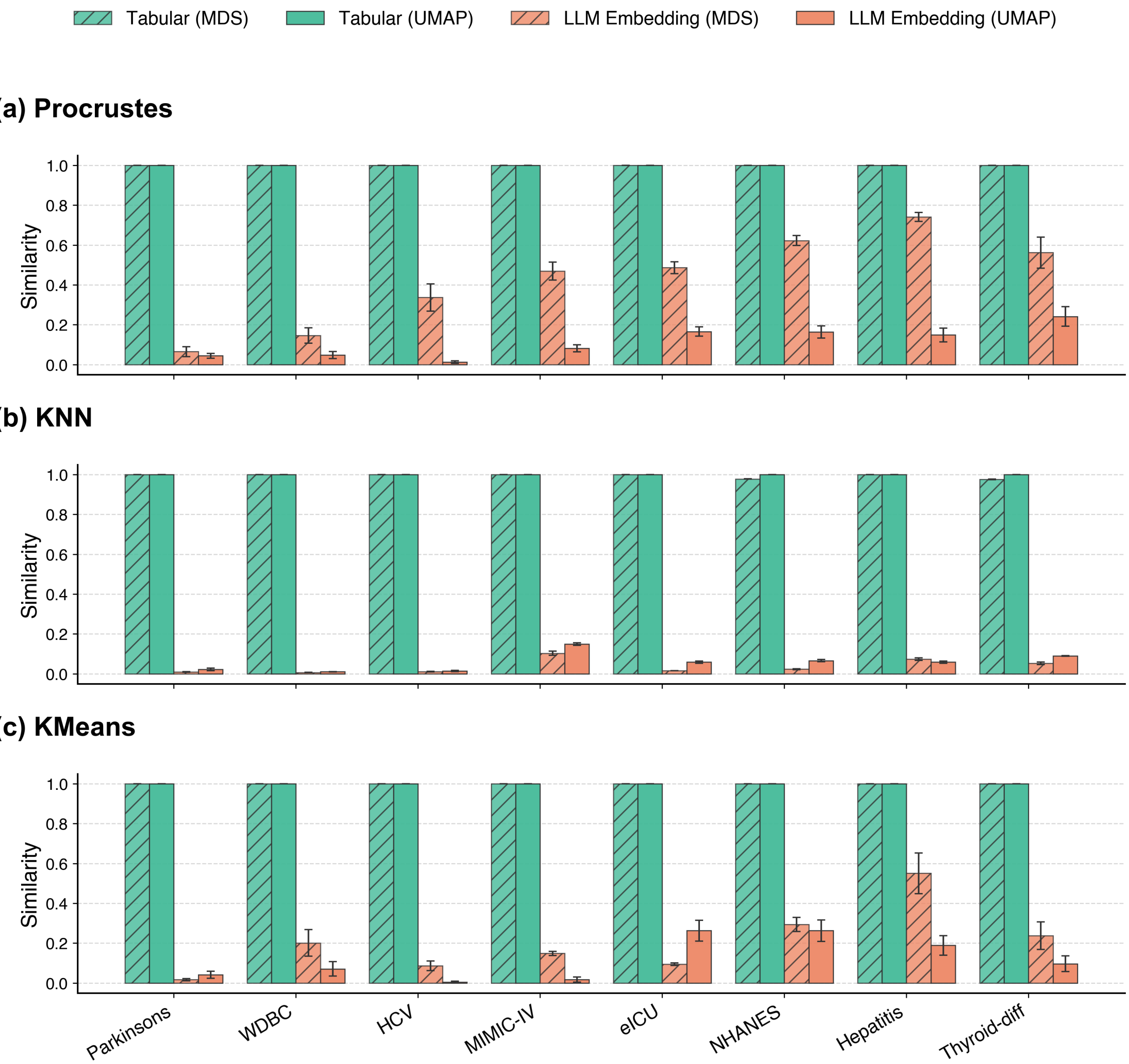

Figure S18: **Dimension reduction stability under permutation of feature order when a JSON serialization template is used for the embedding-based approach.** For each dataset, the columns of the tabular data were randomly permuted, and the resulting low-dimensional representation was compared with that obtained from the original unpermuted data. Stability was quantified using (a) Procrustes similarity, (b) KNN similarity, and (c) KMeans similarity. Higher values indicate greater stability, with 1 corresponding to perfect agreement and values near 0 indicating little meaningful agreement. For the LLM embedding-based approach, each observation was serialized using a JSON format before computing embeddings; results for the direct tabular approach are shown for comparison. Both approaches were combined with either MDS or UMAP.

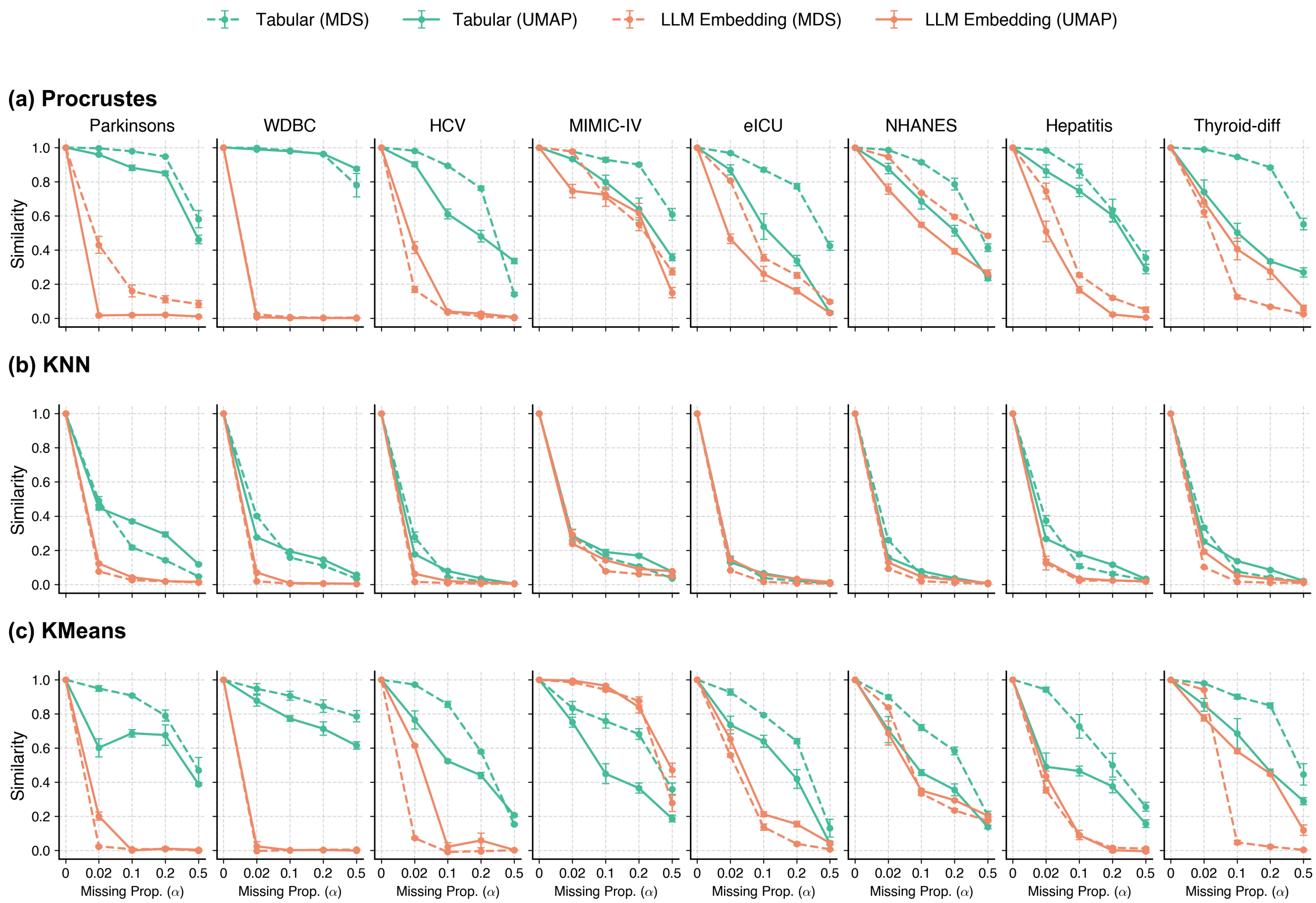

Figure S19: **Dimension reduction stability under missing completely at random (MCAR) when the `all-mpnet-base-v2` embedding model is used.** Additional missing values were introduced independently into each cell with probability α. Stability was assessed by comparing the resulting low-dimensional representation with that obtained from the original data using three complementary measures: (a) Procrustes similarity, (b) KNN similarity, and (c) KMeans similarity. Higher values indicate greater stability, with 1 corresponding to perfect agreement and values near 0 indicating little meaningful agreement. For the embedding-based approach, the open-source `all-mpnet-base-v2` model (Sentence-Transformers) was used in place of OpenAI's API. This model is fully deterministic and produces embeddings of dimension 768. Results for the direct tabular approach are shown for comparison. Both approaches were combined with either MDS or UMAP.

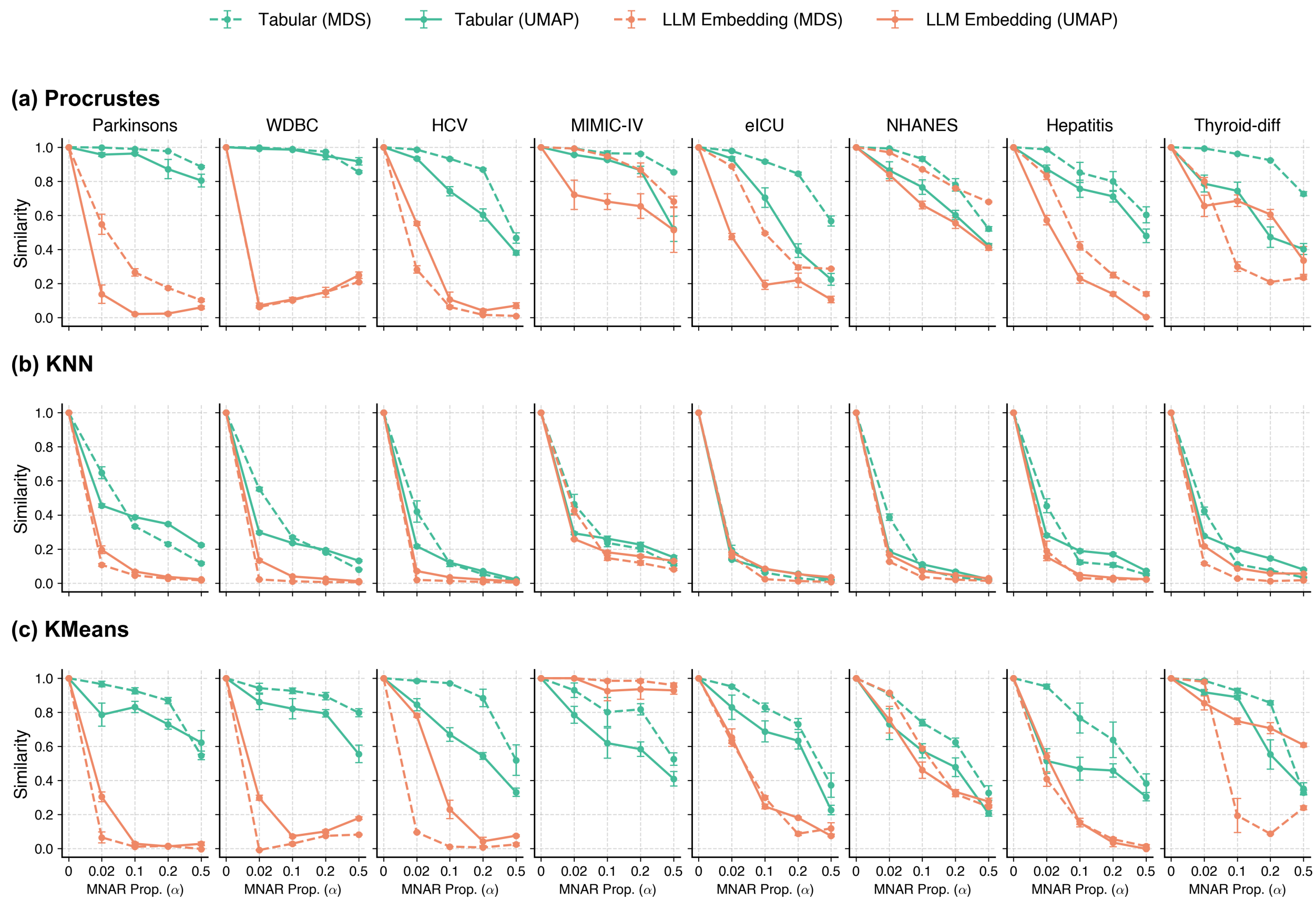

Figure S20: **Dimension reduction stability under missing not at random (MNAR) when the `all-mpnet-base-v2` embedding model is used.** Additional missing values were introduced using a structured missingness mechanism, and the resulting low-dimensional representation was compared with that from the original data. Stability was quantified using (a) Procrustes similarity, (b) KNN similarity, and (c) KMeans similarity. Higher values indicate greater stability, with 1 corresponding to perfect agreement and values near 0 indicating little meaningful agreement. For the embedding-based approach, the open-source `all-mpnet-base-v2` model (Sentence-Transformers) was used in place of OpenAI's API. This model is fully deterministic and produces embeddings of dimension 768. Results for the direct tabular approach are shown for comparison. Both approaches were combined with either MDS or UMAP.

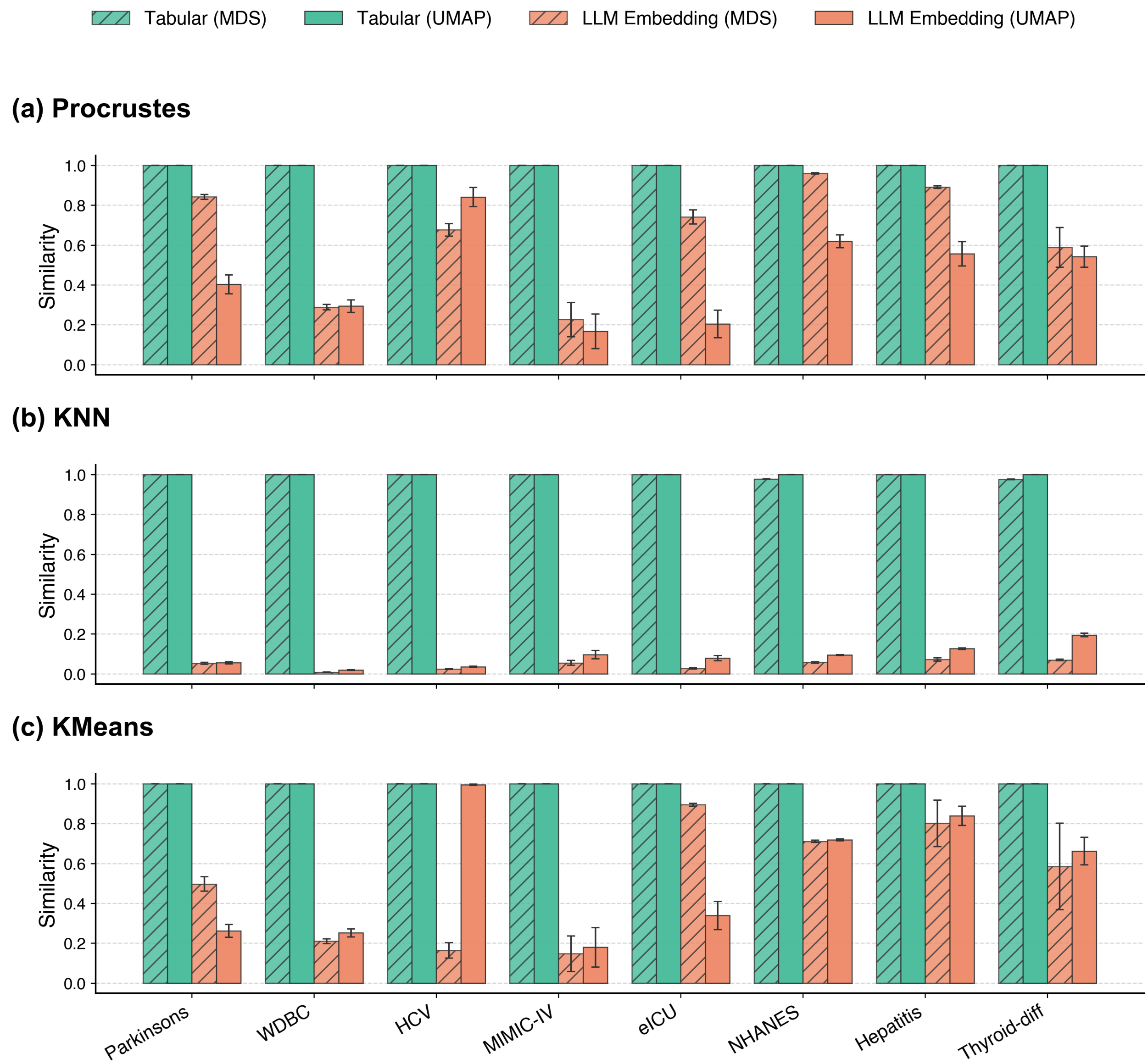

Figure S21: **Dimension reduction stability under permutation of feature order when the `all-mpnet-base-v2` embedding model is used.** For each dataset, the columns of the tabular data were randomly permuted, and the resulting low-dimensional representation was compared with that obtained from the original unpermuted data. Stability was quantified using (a) Procrustes similarity, (b) KNN similarity, and (c) KMeans similarity. Higher values indicate greater stability, with 1 corresponding to perfect agreement and values near 0 indicating little meaningful agreement. For the embedding-based approach, the open-source `all-mpnet-base-v2` model (Sentence-Transformers) was used in place of OpenAI's API. This model is fully deterministic and produces embeddings of dimension 768. Results for the direct tabular approach are shown for comparison. Both approaches were combined with either MDS or UMAP.

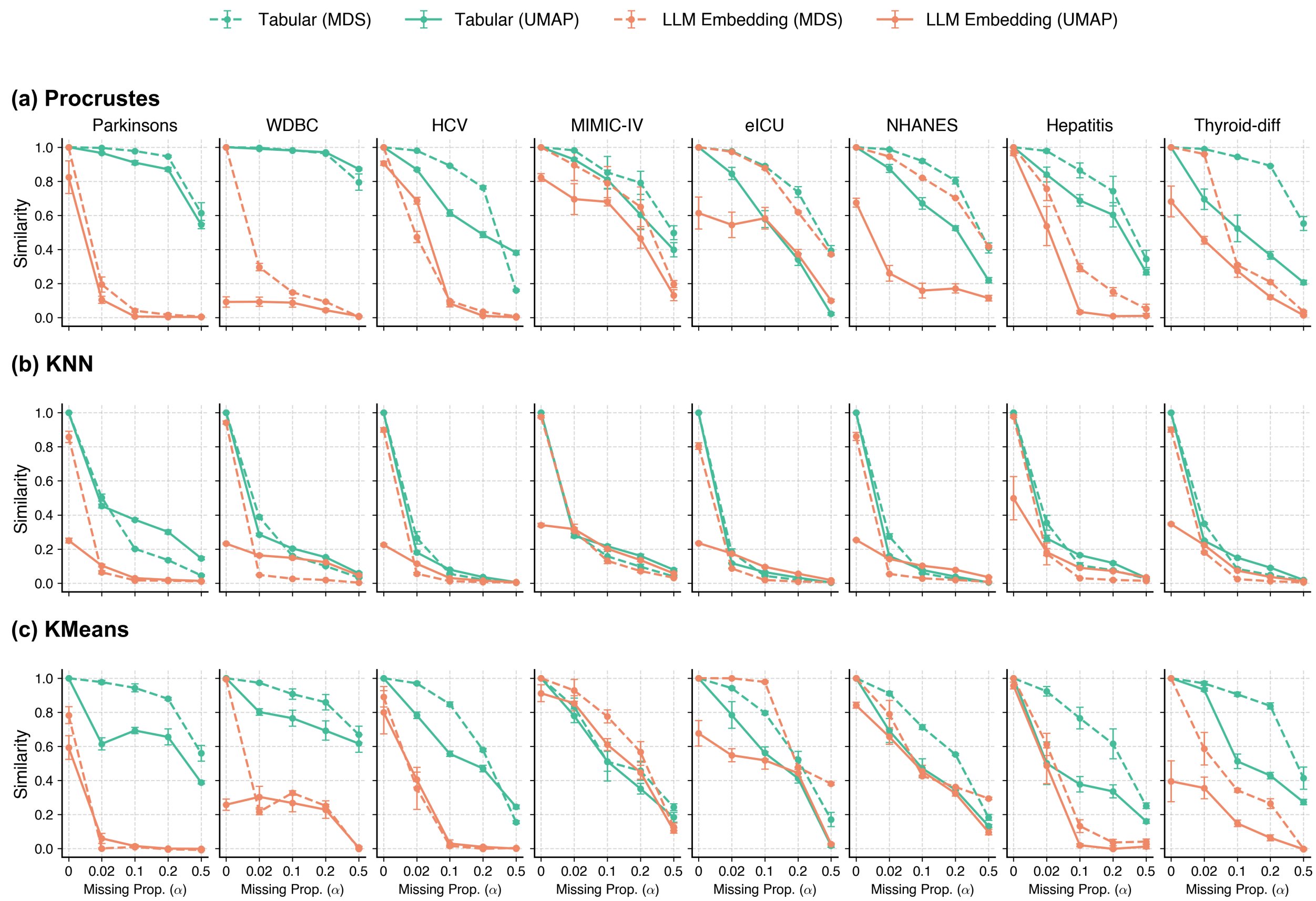

Figure S22: **Dimension reduction stability under missing completely at random (MCAR) when features are sorted alphabetically before serialization.** Additional missing values were introduced independently into each cell with probability α. Stability was assessed by comparing the resulting low-dimensional representation with that obtained from the original data using three complementary measures: (a) Procrustes similarity, (b) KNN similarity, and (c) KMeans similarity. Higher values indicate greater stability, with 1 corresponding to perfect agreement and values near 0 indicating little meaningful agreement. For the LLM embedding-based approach, features were sorted alphabetically by name before serialization to enforce a canonical ordering; results for the direct tabular approach are shown for comparison. Both approaches were combined with either MDS or UMAP.

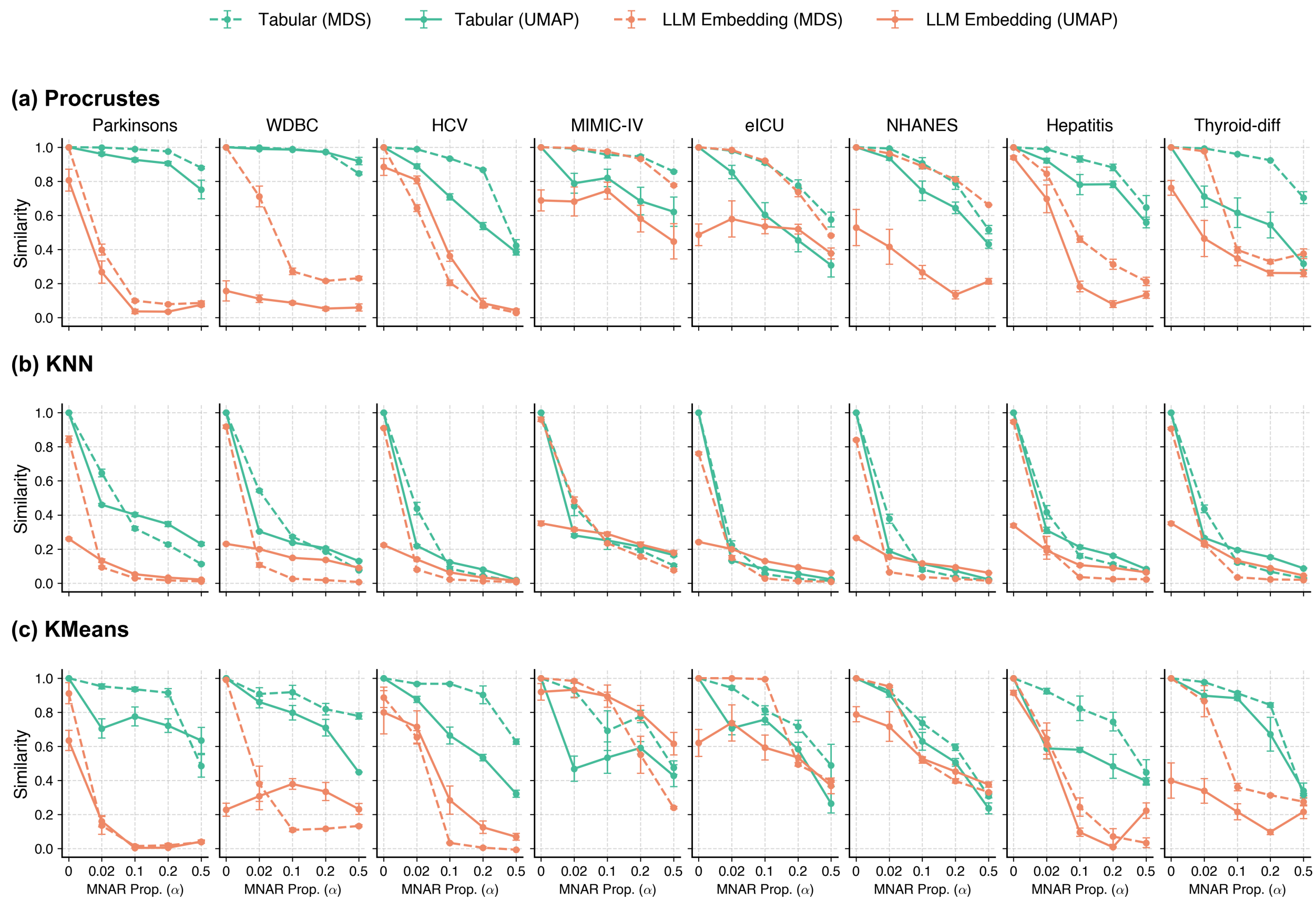

Figure S23: **Dimension reduction stability under missing not at random (MNAR) when features are sorted alphabetically before serialization.** Additional missing values were introduced using a structured missingness mechanism, and the resulting low-dimensional representation was compared with that from the original data. Stability was quantified using (a) Procrustes similarity, (b) KNN similarity, and (c) KMeans similarity. Higher values indicate greater stability, with 1 corresponding to perfect agreement and values near 0 indicating little meaningful agreement. For the LLM embedding-based approach, features were sorted alphabetically by name before serialization to enforce a canonical ordering; results for the direct tabular approach are shown for comparison. Both approaches were combined with either MDS or UMAP.

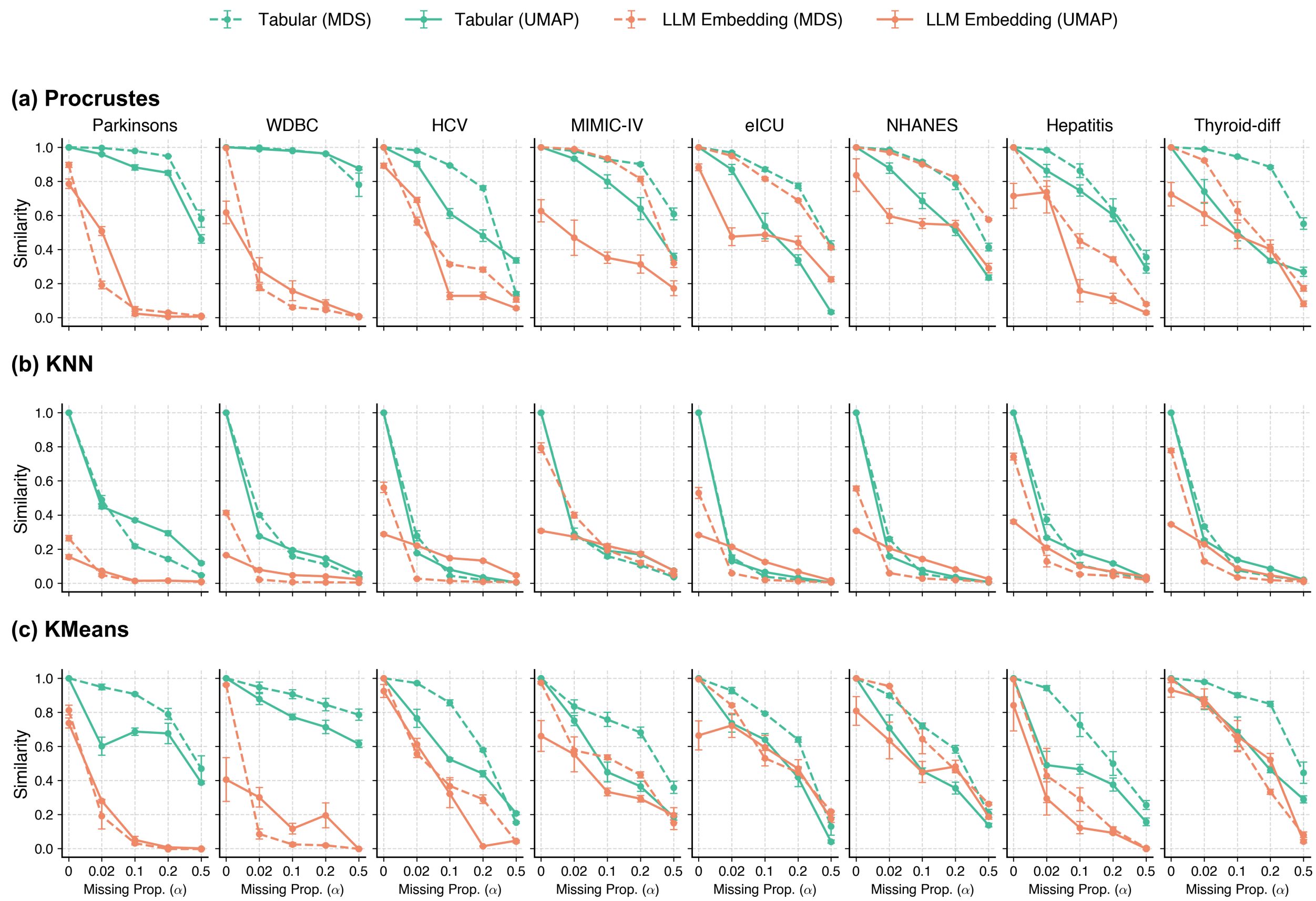

Figure S24: **Dimension reduction stability under missing completely at random (MCAR) when OpenAI's `text-embedding-3-large` model is used for the embedding-based approach.** Additional missing values were introduced independently into each cell with probability α. Stability was assessed by comparing the resulting low-dimensional representation with that obtained from the original data using three complementary measures: (a) Procrustes similarity, (b) KNN similarity, and (c) KMeans similarity. Higher values indicate greater stability, with 1 corresponding to perfect agreement and values near 0 indicating little meaningful agreement. For the LLM embedding-based approach, embeddings were generated using this model; results for the direct tabular approach are shown for comparison. Both approaches were combined with either MDS or UMAP.

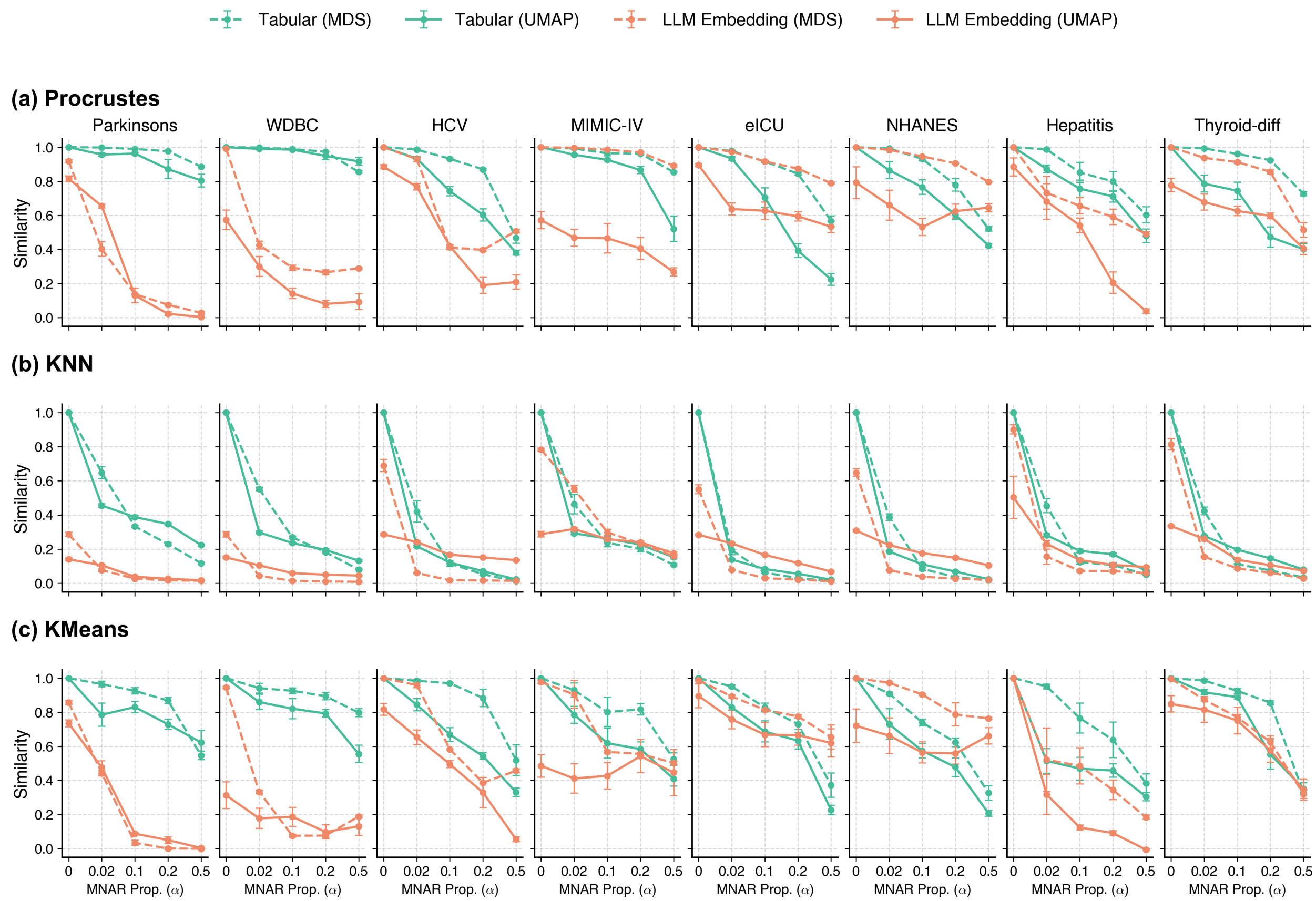

Figure S25: **Dimension reduction stability under missing not at random (MNAR) when OpenAI's `text-embedding-3-large` model is used for the embedding-based approach.** Additional missing values were introduced using a structured missingness mechanism, and the resulting low-dimensional representation was compared with that from the original data. Stability was quantified using (a) Procrustes similarity, (b) KNN similarity, and (c) KMeans similarity. Higher values indicate greater stability, with 1 corresponding to perfect agreement and values near 0 indicating little meaningful agreement. For the LLM embedding-based approach, embeddings were generated using this model; results for the direct tabular approach are shown for comparison. Both approaches were combined with either MDS or UMAP.

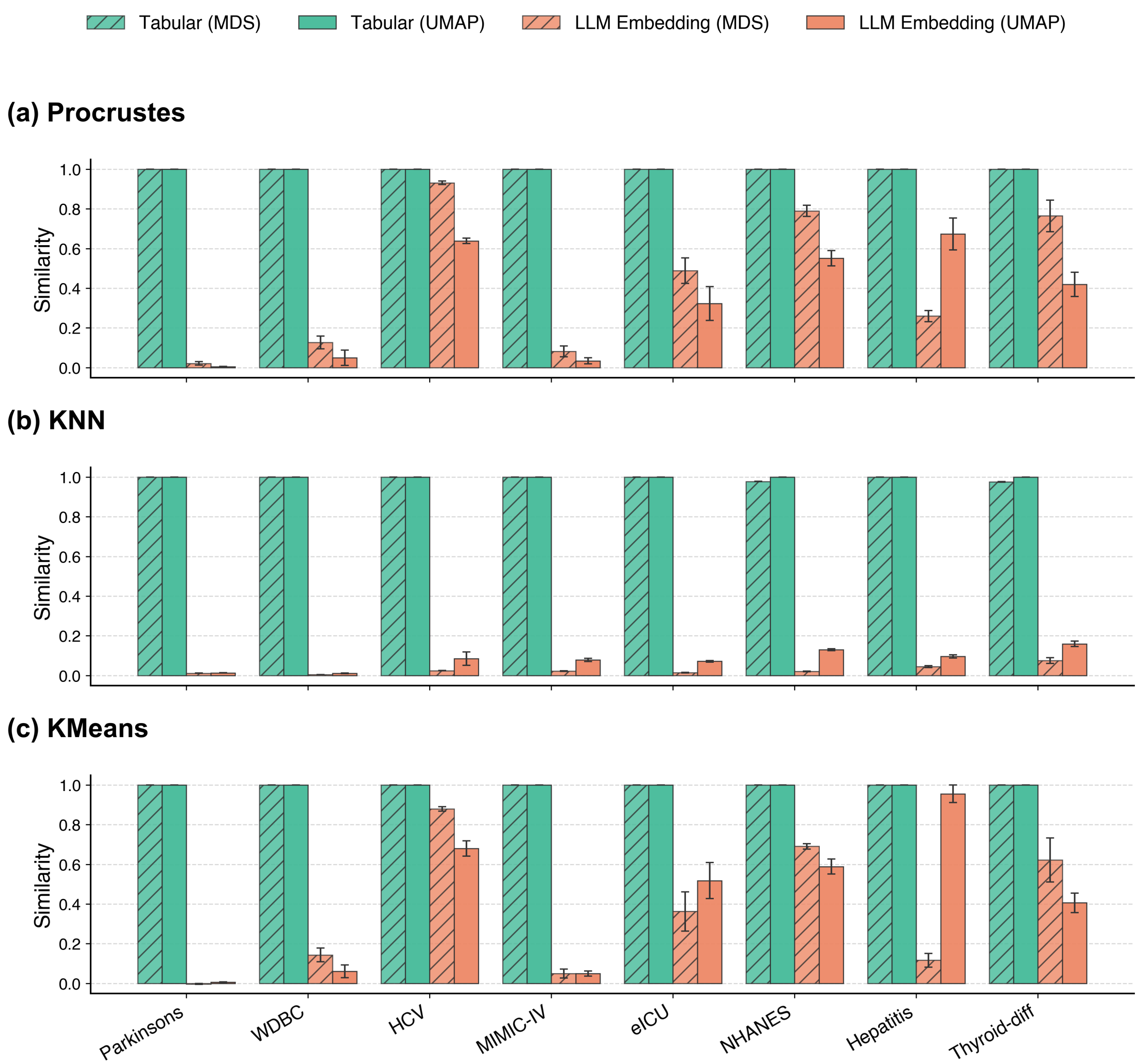

Figure S26: **Dimension reduction stability under permutation of feature order when OpenAI's `text-embedding-3-large` model is used for the embedding-based approach.** For each dataset, the columns of the tabular data were randomly permuted, and the resulting low-dimensional representation was compared with that obtained from the original unpermuted data. Stability was quantified using (a) Procrustes similarity, (b) KNN similarity, and (c) KMeans similarity. Higher values indicate greater stability, with 1 corresponding to perfect agreement and values near 0 indicating little meaningful agreement. For the LLM embedding-based approach, embeddings were generated using this model; results for the direct tabular approach are shown for comparison. Both approaches were combined with either MDS or UMAP.

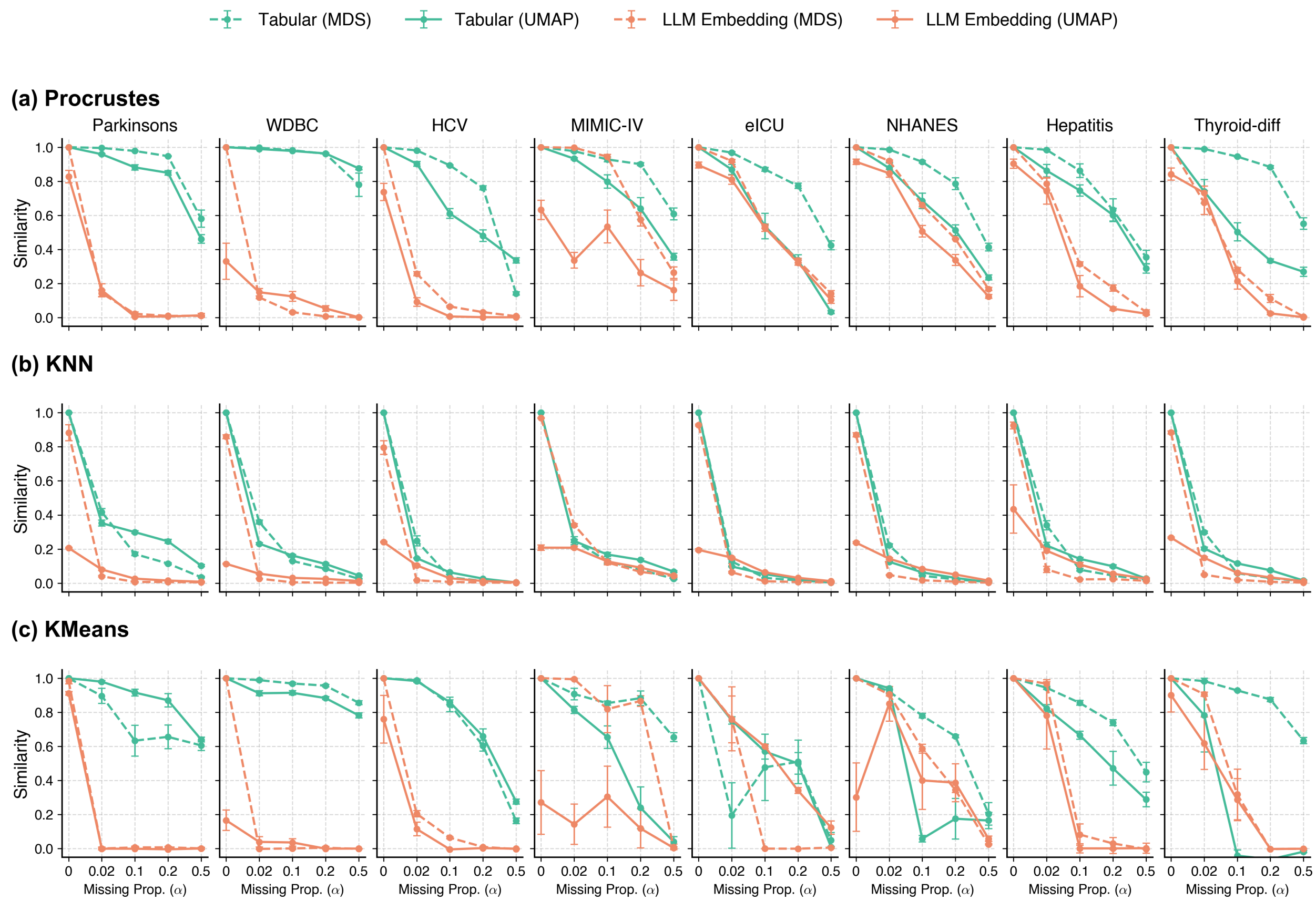

Figure S27: **Dimension reduction stability under missing completely at random (MCAR) when $K = 2$ is used for both KNN similarity and KMeans similarity.** Additional missing values were introduced independently into each cell with probability α. Stability was assessed by comparing the resulting low-dimensional representation with that obtained from the original data using (a) Procrustes similarity, (b) KNN similarity with $K = 2$, and (c) KMeans similarity with $K = 2$. Higher values indicate greater stability, with 1 corresponding to perfect agreement and values near 0 indicating little meaningful agreement. Results are shown for both the LLM embedding-based approach and the direct tabular approach, each combined with either MDS or UMAP.

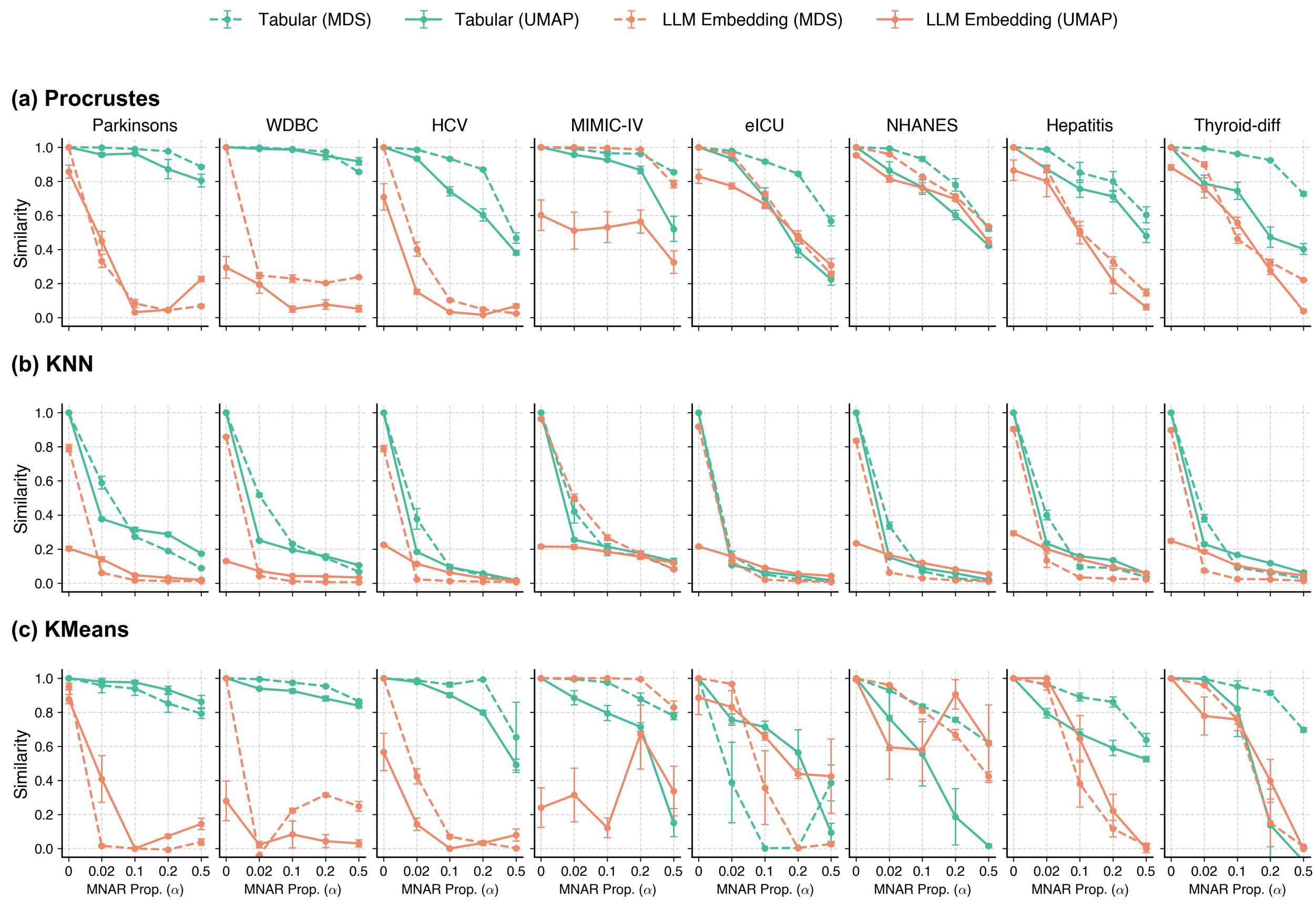

Figure S28: **Dimension reduction stability under missing not at random (MNAR) when $K = 2$ is used for both KNN similarity and KMeans similarity.** Additional missing values were introduced using a structured missingness mechanism, and the resulting low-dimensional representation was compared with that from the original data. Stability was quantified using (a) Procrustes similarity, (b) KNN similarity with $K = 2$, and (c) KMeans similarity with $K = 2$. Higher values indicate greater stability, with 1 corresponding to perfect agreement and values near 0 indicating little meaningful agreement. Results are shown for both the LLM embedding-based approach and the direct tabular approach, each combined with either MDS or UMAP.

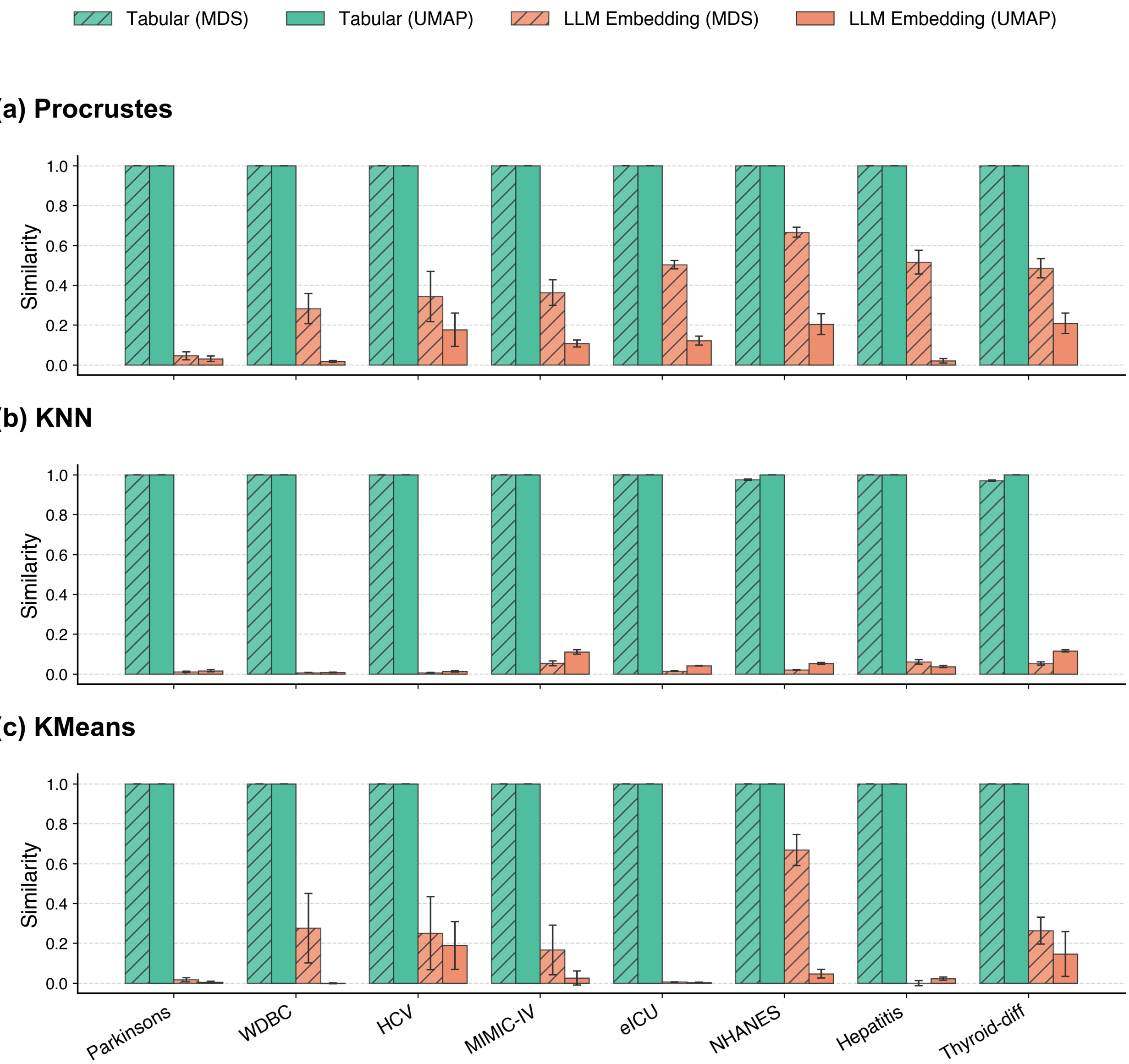

Figure S29: **Dimension reduction stability under permutation of feature order when $K = 2$ is used for both KNN similarity and KMeans similarity.** For each dataset, the columns of the tabular data were randomly permuted, and the resulting low-dimensional representation was compared with that obtained from the original unpermuted data. Stability was quantified using (a) Procrustes similarity, (b) KNN similarity with $K = 2$, and (c) KMeans similarity with $K = 2$. Higher values indicate greater stability, with 1 corresponding to perfect agreement and values near 0 indicating little meaningful agreement. Results are shown for both the LLM embedding-based approach and the direct tabular approach, each combined with either MDS or UMAP.

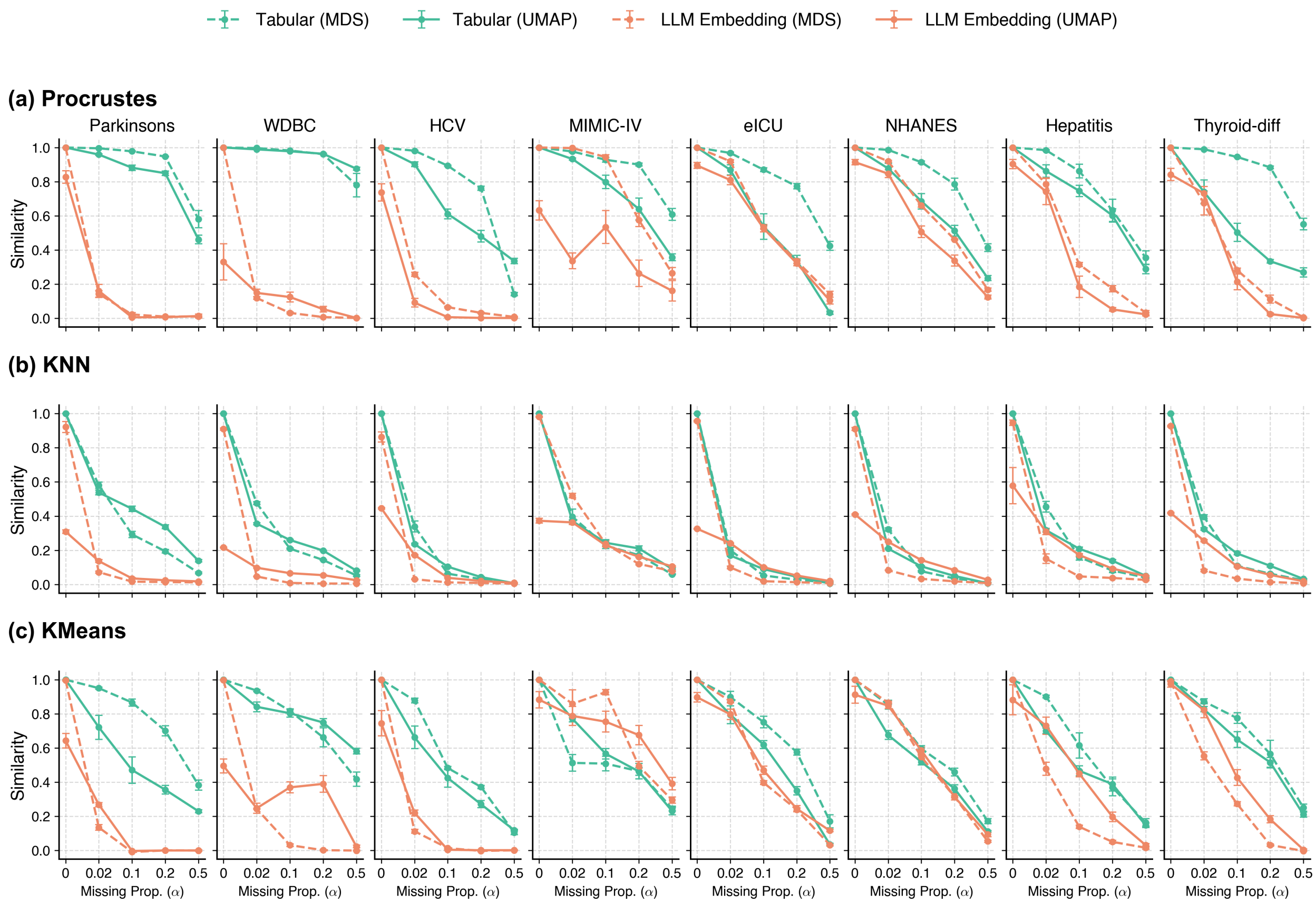

Figure S30: **Dimension reduction stability under missing completely at random (MCAR) when $K = 5$ is used for both KNN similarity and KMeans similarity.** Additional missing values were introduced independently into each cell with probability α. Stability was assessed by comparing the resulting low-dimensional representation with that obtained from the original data using (a) Procrustes similarity, (b) KNN similarity with $K = 5$, and (c) KMeans similarity with $K = 5$. Higher values indicate greater stability, with 1 corresponding to perfect agreement and values near 0 indicating little meaningful agreement. Results are shown for both the LLM embedding-based approach and the direct tabular approach, each combined with either MDS or UMAP.

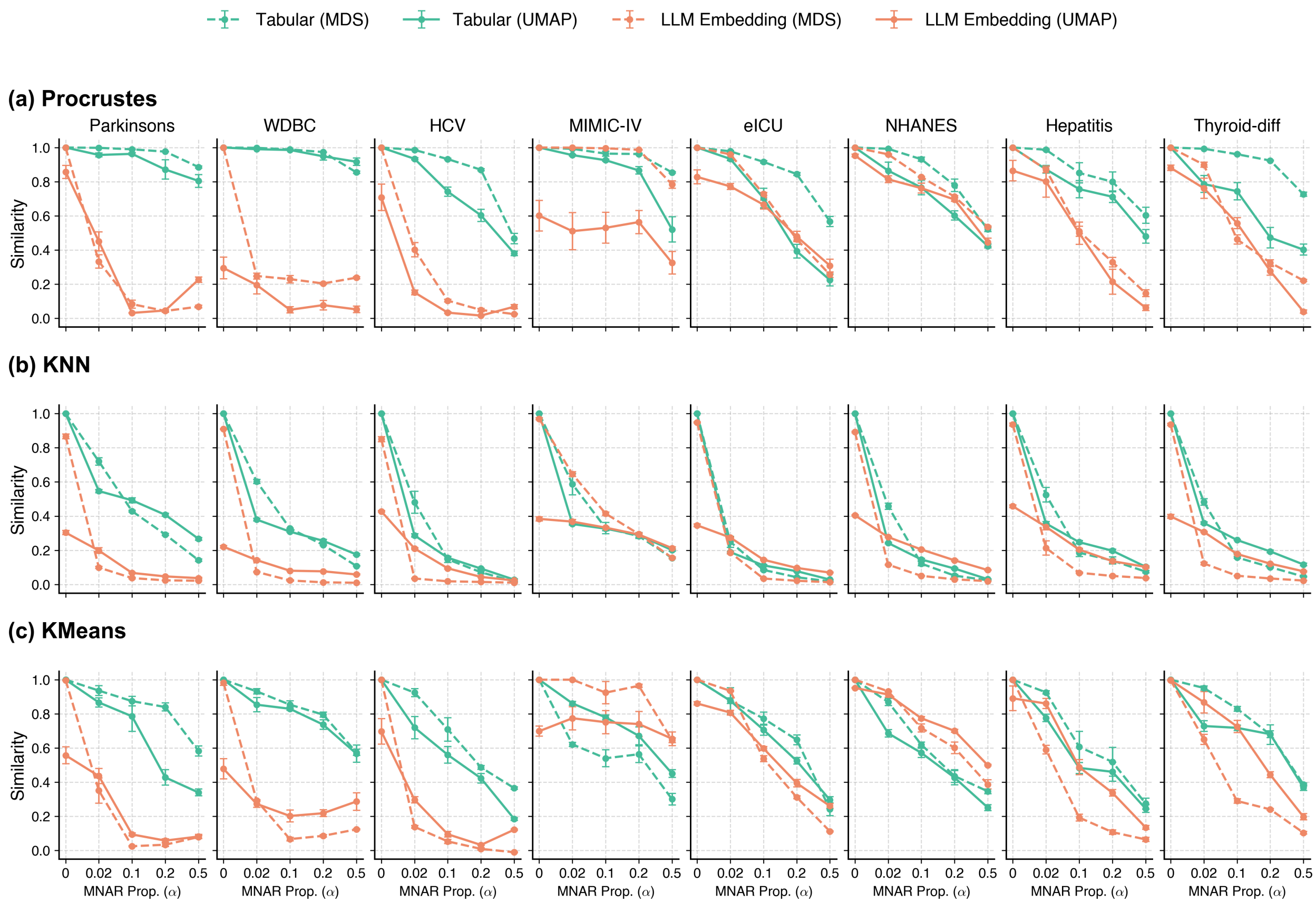

Figure S31: **Dimension reduction stability under missing not at random (MNAR) when $K = 5$ is used for both KNN similarity and KMeans similarity.** Additional missing values were introduced using a structured missingness mechanism, and the resulting low-dimensional representation was compared with that from the original data. Stability was quantified using (a) Procrustes similarity, (b) KNN similarity with $K = 5$, and (c) KMeans similarity with $K = 5$. Higher values indicate greater stability, with 1 corresponding to perfect agreement and values near 0 indicating little meaningful agreement. Results are shown for both the LLM embedding-based approach and the direct tabular approach, each combined with either MDS or UMAP.

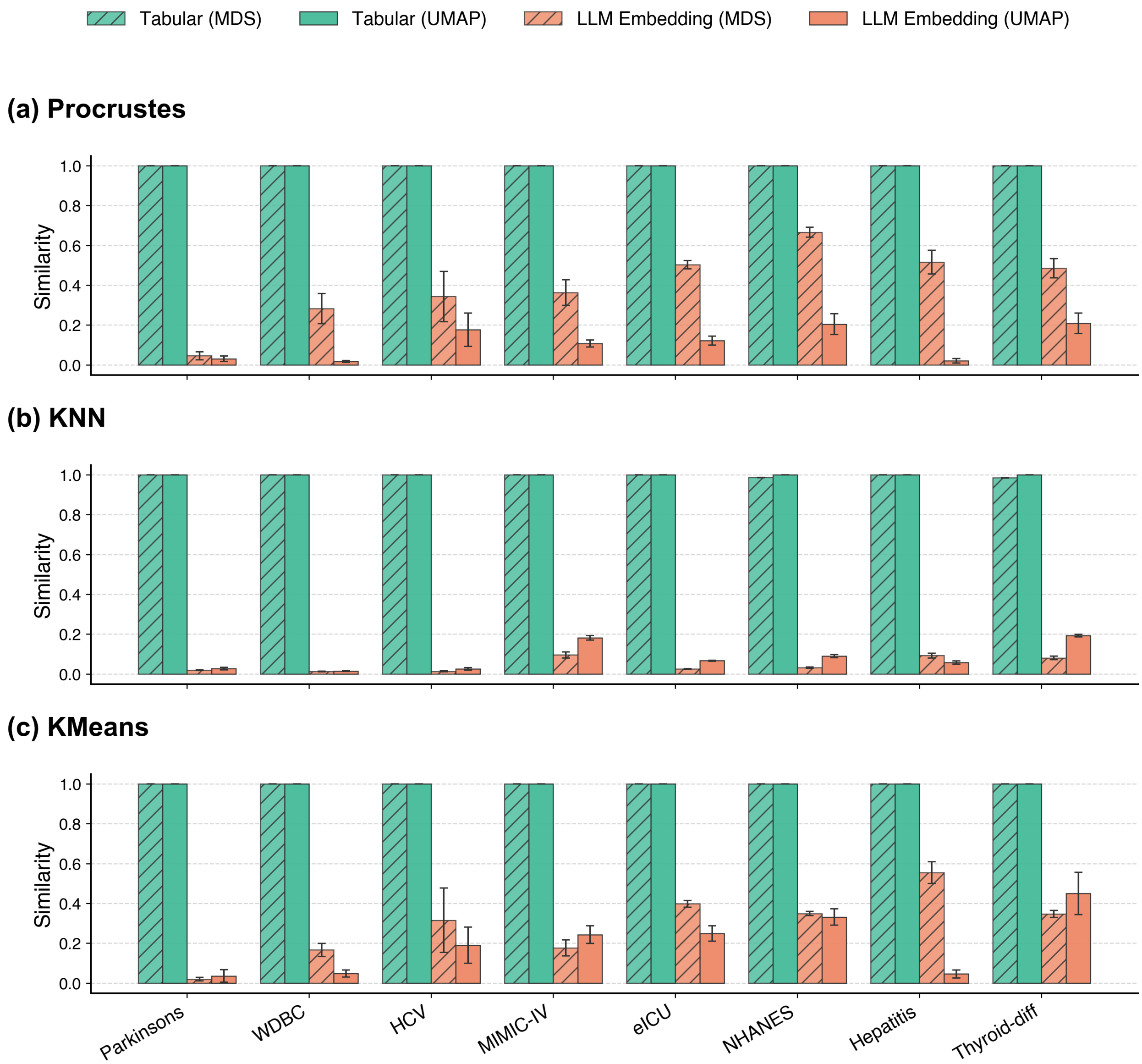

Figure S32: **Dimension reduction stability under permutation of feature order when $K = 5$ is used for both KNN similarity and KMeans similarity.** For each dataset, the columns of the tabular data were randomly permuted, and the resulting low-dimensional representation was compared with that obtained from the original unpermuted data. Stability was quantified using (a) Procrustes similarity, (b) KNN similarity with $K = 5$, and (c) KMeans similarity with $K = 5$. Higher values indicate greater stability, with 1 corresponding to perfect agreement and values near 0 indicating little meaningful agreement. Results are shown for both the LLM embedding-based approach and the direct tabular approach, each combined with either MDS or UMAP.
